# Herbivore diversity effects on Arctic tundra ecosystems: a systematic review

**DOI:** 10.1186/s13750-024-00330-9

**Published:** 2024-03-25

**Authors:** Laura Barbero-Palacios, Isabel C. Barrio, Mariana García Criado, Ilona Kater, Matteo Petit Bon, Tiina H. M. Kolari, Ragnhild Bjørkås, Jonas Trepel, Erick Lundgren, Katrín Björnsdóttir, Bernice C. Hwang, Laura Bartra-Cabré, Mathilde Defourneaux, Jennifer Ramsay, Thomas K. Lameris, A. Joshua Leffler, Janine G. Lock, Mari S. Kuoppamaa, Jeppe A. Kristensen, Anne D. Bjorkman, Isla Myers-Smith, Nicolas Lecomte, Jan C. Axmacher, Olivier Gilg, Michael Den Herder, Emmanuel P. Pagneux, Anna Skarin, Natalia Sokolova, Torben Windirsch, Helen C. Wheeler, Emmanuel Serrano, Tarmo Virtanen, David S. Hik, Elina Kaarlejärvi, James D. M. Speed, Eeva M. Soininen

**Affiliations:** 1 Faculty of Environmental and Forest Sciences, Agricultural University of Iceland, Árleyni 22, Keldnaholt, IS-112 Reykjavík, Iceland; 2 School of GeoSciences, University of Edinburgh, Edinburgh, Scotland EH9 3FF UK; 3 Scott Polar Research Institute, University of Cambridge, Cambridge, UK; 4 Department of Wildland Resources | Quinney College of Natural Resources and Ecology Center, Utah State University, Logan, Utah UT-84322 USA; 5 Department of Geographical and Historical Studies, University of Eastern Finland, P.O. Box 111, 80101 Joensuu, Finland; 6 Centre for Biodiversity Dynamics, Department of Biology, Norwegian University of Science and Technology, 7491 Trondheim, Norway; 7 Center for Ecological Dynamics in a Novel Biosphere (ECONOVO), Section for Ecoinformatics and Biodiversity, Department of Biology, Aarhus University, Aarhus, Denmark; 8 School of Biology and Environmental Science, Faculty of Science, Queensland University of Technology, Brisbane, QLD Australia; 9 Department of Biological and Environmental Sciences, University of Gothenburg, P.O. Box 461, 405 30 Gothenburg, Sweden; 10 Department of Ecology & Environmental Science, Umeå University, KBC-Huset, Linnaeus Väg 6, 901 87 Umeå, Sweden; 11 Department of Ecology, University of Innsbruck, Sternwartestraße 15, 6020 Innsbruck, Austria; 12 School of Life Sciences, Anglia Ruskin University, East Road, Cambridge, CB1 1PT UK; 13 Department of Coastal Systems, Royal Netherlands Institute for Sea Research (NIOZ), Den Burg, The Netherlands; 14 Department of Natural Resource Management, South Dakota State University, Brookings, SD USA; 15 Arctic Centre, University of Lapland, Rovaniemi, Finland; 16 School of Environmental Sciences, University of Liverpool, Liverpool, UK; 17 Environmental Change Institute, School of Geography and the Environment, University of Oxford, Oxford, UK; 18 Department of Forest and Conservation Science, Faculty of Forestry, University of British Columbia, 3041-2424 Main Mall, Vancouver, BC V6T 1Z4 Canada; 19 Canada Research Chair in Polar and Boreal Ecology, Department of Biology and Centre d’Études Nordiques, Université de Moncton, Moncton, E1A 3E9 Canada; 20 Department of Geography, UCL, University College London, London, UK; 21 UMR 6249 Chrono-Environnement, CNRS, Université de Franche-Comté, 25000 Besançon, France; 22 Groupe de Recherche en Ecologie Arctique, 21440 Francheville, France; 23 European Forest Institute, 80100 Joensuu, Finland; 24 Department of Animal Nutrition and Management, Swedish University of Agricultural Sciences, Uppsala, Sweden; 25 Arctic Research Station of Institute of Plant and Animal Ecology Ural Branch, Russian Academy of Sciences, Labytnangi, Russia; 26 Permafrost Research Section, Alfred Wegener Institute Helmholtz Centre for Polar and Marine Research, Potsdam, Germany; 27 Wildlife Ecology & Health Group (WE&H), Servei d’Ecopatologia de Fauna Salvatje (SEFaS), Department de Medicina I Cirurgia Animals, Universitat Autònoma de Barcelona (UAB), Bellaterra, Spain; 28 Ecosystems and Environment Research Programme, Faculty of Biological and Environmental Sciences, University of Helsinki, Helsinki, Finland; 29 Department of Biological Sciences, Simon Fraser University, Burnaby, BC V5A 1S6 Canada; 30 Research Centre for Ecological Change, Organismal and Evolutionary Biology Research Programme, Faculty of Biological and Environmental Sciences, University of Helsinki, Helsinki, Finland; 31 Department of Natural History, NTNU University Museum, Norwegian University of Science and Technology, 7491 Trondheim, Norway; 32 Department of Arctic and Marine Biology, UiT The Arctic University of Norway, Tromsø, Norway

**Keywords:** Herbivore assemblage, Body size, Browsing, Grazing, Defoliation, Ecosystem function, Plant-herbivore-soil interaction, Species richness

## Abstract

**Background:**

Northern ecosystems are strongly influenced by herbivores that differ in their impacts on the ecosystem. Yet the role of herbivore diversity in shaping the structure and functioning of tundra ecosystems has been overlooked. With climate and land-use changes causing rapid shifts in Arctic species assemblages, a better understanding of the consequences of herbivore diversity changes for tundra ecosystem functioning is urgently needed. This systematic review synthesizes available evidence on the effects of herbivore diversity on different processes, functions, and properties of tundra ecosystems.

**Methods:**

Following a published protocol, our systematic review combined primary field studies retrieved from bibliographic databases, search engines and specialist websites that compared tundra ecosystem responses to different levels of vertebrate and invertebrate herbivore diversity. We used the number of functional groups of herbivores (i.e., functional group richness) as a measure of the diversity of the herbivore assemblage. We screened titles, abstracts, and full texts of studies using pre-defined eligibility criteria. We critically appraised the validity of the studies, tested the influence of different moderators, and conducted sensitivity analyses. Quantitative synthesis (i.e., calculation of effect sizes) was performed for ecosystem responses reported by at least five articles and meta-regressions including the effects of potential modifiers for those reported by at least 10 articles.

**Review findings:**

The literature searches retrieved 5944 articles. After screening titles, abstracts, and full texts, 201 articles including 3713 studies (i.e., individual comparisons) were deemed relevant for the systematic review, with 2844 of these studies included in quantitative syntheses. The available evidence base on the effects of herbivore diversity on tundra ecosystems is concentrated around well-established research locations and focuses mainly on the impacts of vertebrate herbivores on vegetation. Overall, greater herbivore diversity led to increased abundance of feeding marks by herbivores and soil temperature, and to reduced total abundance of plants, graminoids, forbs, and litter, plant leaf size, plant height, and moss depth, but the effects of herbivore diversity were difficult to tease apart from those of excluding vertebrate herbivores. The effects of different functional groups of herbivores on graminoid and lichen abundance compensated each other, leading to no net effects when herbivore effects were combined. In turn, smaller herbivores and large-bodied herbivores only reduced plant height when occurring together but not when occurring separately. Greater herbivore diversity increased plant diversity in graminoid tundra but not in other habitat types.

**Conclusions:**

This systematic review underscores the importance of herbivore diversity in shaping the structure and function of Arctic ecosystems, with different functional groups of herbivores exerting additive or compensatory effects that can be modulated by environmental conditions. Still, many challenges remain to fully understand the complex impacts of herbivore diversity on tundra ecosystems. Future studies should explicitly address the role of herbivore diversity beyond presence-absence, targeting a broader range of ecosystem responses and explicitly including invertebrate herbivores. A better understanding of the role of herbivore diversity will enhance our ability to predict whether and where shifts in herbivore assemblages might mitigate or further amplify the impacts of environmental change on Arctic ecosystems.

**Supplementary Information:**

The online version contains supplementary material available at 10.1186/s13750-024-00330-9.

## Background

Herbivores play a pivotal role in tundra ecosystem dynamics, influencing multiple ecosystem processes, functions, and properties. For example, Arctic herbivores influence tundra carbon and nutrient dynamics through their consumptive and non-consumptive effects [1]. Through selective foraging on specific plant species and the associated changes in plant community composition, herbivores drive plant-soil feedbacks and can accelerate or decelerate rates of primary production, litter decomposition, and nutrient cycling in tundra [2]. Herbivores also impact competitive interactions between above- and below-ground organisms, thereby influencing the composition and functioning of plant, animal, and soil microbial communities [3].

**Fig. 1:**
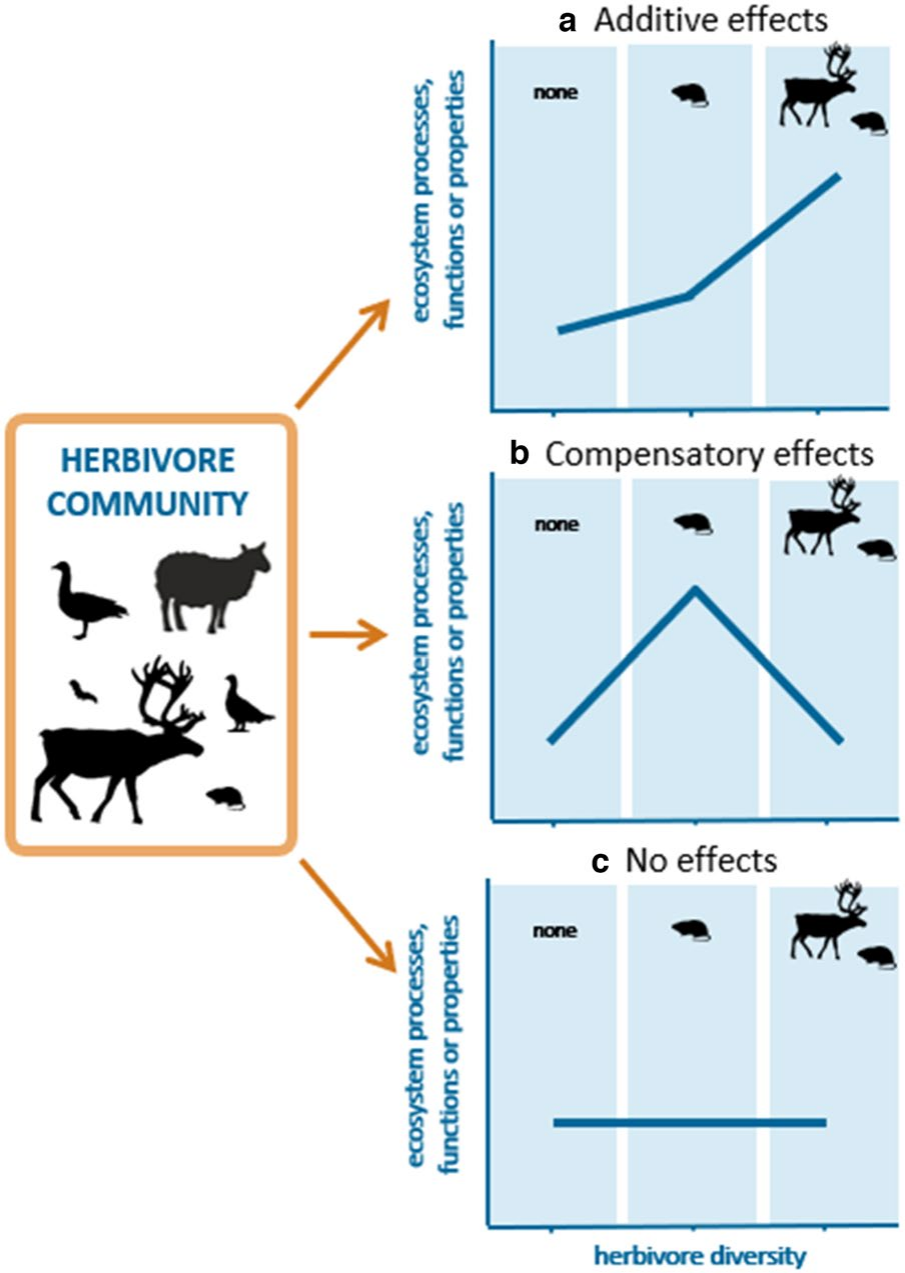
The diversity of the herbivore community (expressed as richness of functional groups of herbivores) can have different effects on tundra ecosystem dynamics (i.e., processes, functions, or properties of tundra ecosystems). These effects can be: **a** additive, where increasing diversity of herbivores leads to strong directional changes in the outcome variable; **b** compensatory, where the effects of different functional groups of herbivores can compensate each other; or **c** no effect, where the outcome variable is not affected by differences in herbivore diversity. We expect the effects of multiple herbivores on ecosystem dynamics to be greater than the effects of single groups of herbivores, although the slope and direction of this relationship can change depending on the response being considered

In the tundra, an array of herbivore species coexists, ranging from invertebrates and small mammals to migratory birds and large ungulates [4]. Differences in body size, energy requirements, digestive physiology, diet choices, behaviour, and population dynamics of these herbivores lead to contrasting effects on ecosystems [5, 6]. In addition, herbivore species rarely occur alone, but in diverse assemblages composed by multiple species. In cases where herbivore groups are functionally similar, the combined effects of different herbivores can add up (i.e., additive effects; Fig. 1a), for example when herbivores consume the same plant species, leading to directional effects that are stronger than those predicted for each group of herbivores separately [7–9]. In such cases, ecosystem effects are often proportional to herbivore abundance. Alternatively, when herbivores are functionally dissimilar, their effects can compensate each other (i.e., compensatory effects; Fig. 1b), for example through consumption of different, competing plant species [10]. In such cases, diverse herbivore assemblages can consume more plant biomass with only a small net effect on plant community composition [11]. Here, one might expect a decoupling between abundance and ecosystem effect. Despite the extensive literature on herbivory in the Arctic [12], there has been no comprehensive attempt to synthesize the impacts of herbivore diversity on tundra ecosystems.

Tundra herbivore communities are dynamic, and future changes to these communities could impact the role of herbivores in ecosystem functioning. In the Pleistocene, tundra herbivore communities were more diverse than in the present day [13]. The loss of key large herbivores in high-latitude regions at the end of the Pleistocene has been linked to widespread ecosystem-level shifts from forb-dominated steppes to shrub-dominated tundra [14]. While contemporary herbivore communities are less diverse than in the Pleistocene [13], they can still drive vegetation shifts [15–17]. However, a more diverse herbivore community would be more likely to drive such shifts [13, 18].

Ongoing environmental change further influences the composition of herbivore communities [5]. Currently, vertebrate herbivore diversity strongly declines northwards across the tundra biome [19], suggesting that climate warming could substantially reshape these communities. Indeed, warming trends have already been linked to the northward expansion of boreal species, i.e., the ‘borealization’ of Arctic herbivore communities [19, 20], and range shifts of Arctic species [21, 22]. Conversely, some herbivores such as caribou (*Rangifer tarandus*) have experienced recent population declines that are potentially linked to global change drivers [23]. If tundra herbivore communities were to either increase or decrease in diversity under projected climate change, this could drive further shifts in tundra ecosystems [13, 18]. Therefore, a greater understanding of the impacts of herbivore diversity across the tundra biome is crucial to predict how future herbivore assemblages may restructure tundra environments.

This systematic review aims to evaluate the effects of vertebrate and invertebrate herbivore diversity on tundra ecosystem dynamics. As a measure of herbivore diversity, we use functional group richness, i.e., the number of herbivore functional groups as defined in [5]. We expect that increased herbivore diversity will influence ecosystem dynamics, but that the strength and direction of these effects may differ depending on the ecosystem process, function or property being considered (Fig. 1). In some cases, the effects of different herbivore groups may be additive or compensatory, or alternatively, changes in herbivore diversity may have no effect.

Our systematic review follows the work on a systematic map of studies on the effects of herbivores on tundra vegetation [12]. Similar to the systematic map, this review is authored by a large number of scientists working on Arctic herbivory, conservation and environmental management agencies that represent the main stakeholder group for the topic. An open call for collaboration through relevant research and professional networks (i.e., the Herbivory Network https://herbivory.lbhi.is/ and UArctic https://www.uarctic.org/) was launched to ensure inclusiveness and active engagement of key participants.

### Objective of the review

The main objective of the systematic review is to synthesize the effects of herbivore diversity on tundra ecosystems, with a particular focus on the role of different herbivore assemblages, rather than individual species, in influencing ecosystem dynamics. This review includes studies assessing the effects of at least two contrasting levels of herbivore diversity on various ecological processes, functions, and properties of tundra ecosystems [24].

*Primary question.* What are the effects of herbivore diversity on processes, functions, and properties of tundra ecosystems?

Components of the primary question:

- *Population:* terrestrial Arctic ecosystems, including the tundra-boreal forest ecotone
- *Exposure:* herbivory, including physical disturbance (e.g., trampling), and fertilization (e.g., dung deposition) effects
- *Comparator:* contrasting herbivore diversity (richness of functional groups of herbivores)
- *Outcome:* measured ecological processes, functions, and properties in response to herbivory

## Methods

This systematic review adhered to the Guidelines and Standards for Systematic Reviews of the Collaboration for Environmental Evidence [25] and the RepOrting standards for Systematic Evidence Syntheses (ROSES [26]); Additional file 1. The systematic review builds on a previous systematic map on the effects of herbivory on Arctic vegetation [12], which identified a substantial number of studies investigating the effect of several herbivores on vegetation, suggesting that a systematic review on this topic was possible. In the present review, we extend the effects of herbivores to include non-vegetation functions, processes and properties in tundra ecosystems. The methods of this review follow a published systematic review protocol [24] and the recently published practical guide for conducting quantitative syntheses in environmental sciences [27]. Full details on literature searches and raw data coded are provided in Additional file 2 and Additional file 3.

### Deviations from the protocol

Minor deviations from the original protocol [24] were necessary. The template used during data coding and study validity assessment (see Additional file 5 in [24]) was adjusted to include additional details on the reviewer coding the study (i.e., reviewer ID). Where relevant, we also included information on the reviewer who edited and/or updated the coding data. Details about study validity assessment and its repeatability, and information on whether studies were included or not in the quantitative synthesis were added to the coding template (Additional file 3). Other minor deviations to specific parts of the workflow are indicated in the corresponding sections below.

### Search for articles

#### Search terms and strings

The full search string used for this systematic review (formatted for Web of Science; search strings for all other sources can be found in Additional file 2 under the databases tab) was the same as in a previously published systematic map on the effects of herbivory on tundra vegetation [12, 28]: (arctic OR subarctic OR tundra) AND (herbivor* OR graz* OR browser OR browsing OR grubb* OR trampl* OR defolia* OR ((invertebrate OR insect) AND (gall* OR mining OR miner)))

This search string did not pose any restriction on the outcome or comparator [24], and was thus broad enough to include all potentially relevant articles for both the systematic map, which focused on the responses of plants to herbivory [12], and for the present systematic review, which addresses the effects of herbivore diversity on tundra ecosystem dynamics.

#### Search sources

Sources were searched between September and November 2021 while the protocol manuscript was still under review. Revisions of the protocol did not affect the main search strategies, so the search was not updated after publication of the protocol. Searches included three main global search sources: Scopus (article title, abstract, and keyword search), Web of Science Core Collection (topic search), and the first 300 search results in Google Scholar (title search standardized to disregard search history; [24]). We also searched for grey literature in specialist websites, modifying the search string as needed depending on their search functionality. Details on the search engines and databases used, including the date when the search was conducted, and details of institutional subscriptions used to access the global search sources and search options, can be found in Additional file 2.

To complement the bibliographic database searches, we performed a snowballing process [29], i.e., we checked the reference lists of articles included after full text screening to identify any potentially relevant articles that had been overlooked in the original searches [24]. These articles were subjected to the same screening process as the articles retrieved from bibliographic database searches.

#### Search limitations

We applied no restrictions regarding date of publication or document type and included peer-reviewed and grey literature that provided primary data. Searches were conducted in English in the global search sources, and in English together with relevant local languages (Russian, French, Finnish, Swedish, Norwegian, Icelandic, and Danish) in the specialist websites, so that language was not a limiting factor to our search.

#### Search results

Articles and their bibliographic information were compiled into a single database formatted for further article screening (Additional file 2). Duplicate articles were removed manually based on their title and bibliographic details.

### Article screening and study eligibility criteria

#### Screening process

Article screening proceeded in three sequential stages: title, abstract, and full text screening (9, 13, and 19 reviewers participated in each stage, respectively; Additional file 4). To streamline the process of article screening, a decision tree was provided for each stage [24]. These decision trees were designed at the protocol development stage, when potential disagreements between reviewers at different stages of the screening process were discussed, and the decision points in the trees were refined to reduce ambiguity in decision making. When in doubt whether an article should pass to the next screening stage, or when reviewers disagreed, the article was included for screening in the following stage. Reviewers did not assess studies they had authored or co-authored. The same reviewer screened both title and abstract of the articles found through Google Scholar and specialist databases or through snowballing, and these articles were only included in our database if deemed relevant at abstract stage. At the full text stage, we excluded publications for which we did not have access to the full text, either in electronic or paper form. We summarized the inclusion and exclusion process at the different screening stages (title, abstract and full text) using a flow diagram (ROSES diagram; [30]). A list of articles excluded at each stage with reasons for exclusion, as well as a full list of included articles, is provided in Additional file 2.

The repeatability of the screening process at different stages was tested during protocol development [24] and during the development of the systematic review, by measuring the consistency of reviewers to either include or exclude an article at each stage (title, abstract and full text). A total of 3520 titles (out of the 3947 articles in the database after removing duplicates; 89.2%), 204 abstracts (out of the 2050 articles included at the title stage excluding correction/replies; 10%) and 28 full texts (out of 633 articles included at abstract stage, excluding correction/replies; 4.4%) were assessed by two independent reviewers. We calculated percent agreement and Cohen’s Kappa (κ) statistic [31] between reviewers using the R package *irr* [32]. Percent agreement reflects the number of times reviewers agreed divided by the total number of screened articles, while Kappa is a measure of interrater reliability (i.e., the extent to which two or more individuals agree; [32]) that incorporates chance agreement between reviewers.

Consistency among reviewers to exclude an article at the title screening stage was moderate (77.4%, κ = 0.53, n = 3520 titles), but it increased at abstract screening, where reviewers agreed in 90.2% of the cases (κ = 0.80, n = 204 abstracts). From the 20 articles for which reviewers disagreed, most (n = 13 articles) were cases where one reviewer suggested excluding the article for lack of eligible comparator. These 20 articles were retained for full-text screening, and only five of them were finally included in our database. At the full-text screening stage, reviewers agreed in 96.4% of the cases (κ = 0.92, n = 28). The only case of disagreement stemmed from one reviewer excluding the article because of lack of clear comparator. After discussion between reviewers, this article was finally included in the database.

#### Eligibility criteria

Each article could report on one or several studies, when separate parts of the article differed in terms of outcome, methodological approach or were reported separately for some other reason. For example, when an article reported an outcome separately for different habitats or study sites, we considered them as separate studies. To be eligible, studies had to report primary data and had to include an eligible population, exposure, comparator, outcome, and type of study design.

##### Eligible populations (terrestrial Arctic ecosystems)

Eligible studies had to focus on Arctic terrestrial ecosystems, geographically defined as those included within the Arctic and subarctic region [33, 34]. Geographical coordinates of studies were extracted from the text, maps or place names. To further limit the scope to tundra and the tundra-forest ecotone, we excluded studies conducted clearly in boreal forests or other non-Arctic terrestrial habitats, as described in the article text. Articles could report on studies conducted at several locations including non-Arctic sites, but in those cases only the subset of studies conducted within the Arctic were included, provided that it was possible to separate data from Arctic and non-Arctic sites.

##### Eligible exposure (herbivory)

Eligible studies had to assess the effects of herbivores on tundra ecosystems (e.g., grazing, browsing, trampling, and other effects, such as fertilizing, digging, or grubbing) or experimental simulations of these effects. Herbivores are considered here as multicellular, terrestrial organisms, and studies had to identify the herbivores to some extent, for example at species level or as a broader group (e.g., small mammalian herbivores).

##### Eligible comparator (contrasting herbivore diversity)

Eligible studies had to assess the effect of herbivore diversity by comparing areas or time periods exposed to different numbers of herbivore species or groups of species (or no herbivore species). Herbivore diversity was defined as richness of functional groups of herbivores. Thus, changes in the relative abundance of some herbivores (for example in studies comparing high versus low reindeer grazing intensity or studies comparing peak versus low phase of rodent population cycles) were not considered a contrast in herbivore diversity for the purposes of this systematic review.

##### Eligible outcome (changes in processes, functions, and properties of terrestrial Arctic ecosystems)

Eligible studies measured the effects of herbivory on processes, functions, and properties of Arctic terrestrial ecosystems. To be included, studies had to report analysable primary data for an outcome and had to provide enough information to enable calculation of effect sizes and measures of variability [35]. This information could take different forms, including estimates of means and variability of an outcome variable for the different levels of herbivore diversity, values for the comparison (effect size) or results of a t-test or one-way ANOVA for the comparison between levels of herbivore diversity.

##### Eligible type of study design

Eligible studies had to be primary field studies (observational or experimental) comparing ecosystem processes, functions and properties in areas or time periods with different levels of herbivore diversity. As such, modelling studies were excluded because they do not provide primary field data. Similarly, greenhouse experiments were excluded because they restrict access of natural herbivores, but we included common garden experiments [36] where herbivores had free access to the experimental areas.

##### Additional criteria

We excluded articles that were not in a suitable format, such as corrections of published articles, raw datasets, conference abstracts, or presentation slides (cf. “unsuitable text types”). Following the strategy of [12], one reviewer (JGL) assessed potential redundancy of studies after all studies had been coded, by checking for potential data overlap in studies conducted within 50 km of the reported geographical coordinates of any other study. We excluded studies that reported data presented in another study, included only the study that reported the longest time series. For chapters of MSc and PhD theses that were published as separate articles, we included only the published peer-reviewed versions.

### Study validity assessment

All the studies fulfilling the eligibility criteria described above were critically appraised for internal validity (i.e., risk of bias and confounding factors) and external validity (i.e., generalisability of results). Critical appraisal was based on seven criteria [37]: the presence of confounding variables, post-intervention or exposure biases, misclassified comparison variables (in the case of observational studies) or performance biases (in the case of experimental studies), detection biases, and risks of bias in outcome reporting or assessment. Studies were classified as having high, medium, or low susceptibility to bias for each of these criteria by the reviewers coding and extracting data from the study. Reviewers followed a decision tree based on the guidelines of [37] adapted to our study question, to assess the risk of bias for each criterion (see Additional file 4 in [24]).

An overall score of risk of bias was also given to each study, considering a high overall risk of bias when at least one criterion was assessed as high, a medium overall risk of bias when at least one criterion was assessed as medium and no criterion was assessed as high, and low overall risk of bias when all criteria were assessed as low [37]. The repeatability of the study validity assessment was tested by measuring the consistency of reviewers in classifying each criterion. A total of 19 reviewers were involved in coding and data extraction, and assessment of study validity (Table S4.1 in Additional file 4). One reviewer (IK) independently assessed a random subset of 10% of the articles (n = 21 articles, corresponding to 856 studies or 23.1% of the database) previously assessed by another reviewer. The results of the critical appraisal are provided for all included studies as part of the coded data in Additional file 3. Overall risk of bias was included as a moderator in analyses to assess the influence of study validity on synthesis results (see Reasons for heterogeneity and selection of potential effect modifiers).

Consistency among reviewers in scoring study validity (856 studies) was substantial for overall score of risk of bias (89.4% agreement, Cohen’s kappa κ = 0.67). When looking at the criteria separately, the agreement between reviewers was substantial for the risk of misclassified comparison or performance bias (criteria 3 and 4; 87.9% agreement, Cohen’s kappa κ = 0.67) and moderate for other criteria (agreement between 75.6 and 83.1%, κ between 0.44 and 0.55; see Additional file 4).

### Data coding and extraction strategy

For each eligible article, we extracted information using a data coding template developed as part of the protocol (see Additional file 5 in [24]). Information was recorded separately for each study. If studies reported repeated measurements over the growing season, we extracted data from the peak of the growing season (i.e., late July-early August in Arctic terrestrial ecosystems), and if they reported repeated annual measurements, we extracted data from the last year of measurements. When a study reported several comparisons of herbivore diversity levels, for example in studies using size-selective exclosures, where herbivores of different sizes are excluded sequentially, pairwise comparisons were extracted as separate studies.

When studies provided more than one type of data for outcome variables (for example, when both the mean and variance of the outcome variable for high and low herbivore diversity, and the statistical test for the comparison were reported), all data were extracted. We extracted data from the text and tables, or from graphs using image analysis software (ImageJ; [38]) when relevant information was not included in the text or tables. In a few cases (n = 10 articles), authors of relevant articles were contacted to request access to unpublished primary data or to ask for confirmation of missing or unclear information. The extracted data records can be found in Additional file 3.

Using the coding template ensured consistency during the extraction of raw data from the studies [24]. In addition, we ran an online and a hybrid workshop, where reviewers were trained in the data extraction process, by coding one article together and working in smaller groups. Once the database of all coded studies was assembled, two reviewers (LBP and ICB) checked all the coded data for accuracy, corrected obvious errors in the database, identified extreme outliers using diagnostic plots and checked them against the original article, and edited possible dissimilarities in spelling. For outcome variables, detailed descriptions were replaced by shorter, more general variable descriptions to ensure consistency in outcome variable names (see outcomes in Additional file 3). These steps made unnecessary the repeatability assessment of the data extraction process proposed in the original protocol.

The outcome variables measured in each study were grouped for summary purposes into larger categories prior to the synthesis (Table S4.2 in Additional file 4). Plant abundance was by far the most reported outcome variable (reported by 111 articles and 1246 studies; 33.6% of studies). Since different functional groups of plants differ in their responses to herbivores [39], we further split this outcome variable into plant functional groups (for details on plant functional groups used, see Table S4.3 in Additional file 4).

Because of inconsistencies across studies in reporting the taxonomic identity of herbivores, we grouped herbivores into functional groups based on [5] (Table S4.4 in Additional file 4): F1 are herbivores associated with limnic-habitats, migrating outside the Arctic for winter, with undifferentiated guts and for which graminoids are an important diet component (waterfowl; paragon *Anser anser*); F2 are non-migratory, burrowing herbivores with hindgut fermenting digestive physiology (paragon *Synaptomys borealis*); and F3 are large-bodied facultative-generalist herbivores for which shrubs and lichens are an important diet component (paragon *Lepus timidus*). Given the broad groups defined for vertebrate herbivores, invertebrate herbivores were kept as a single, separate group irrespective of their feeding group [40]. As an alternative, we grouped herbivores based on their body size, an important trait that determines herbivore impacts on ecosystems [41, 42]: invertebrate herbivores, small, medium, and large vertebrate herbivores. Results of these analyses were broadly consistent to those of functional groups and are presented in Figure S4.2 in Additional file 4.

After reclassifying the herbivores into broader groups, we calculated herbivore diversity contrasts for each study as the difference in herbivore groups between the high and low herbivore diversity levels reported by the study, in terms of the number of herbivore groups or the identity of those groups (Table 1). For example, a study comparing areas where waterfowl (F1) and large herbivores (F3) are present (high herbivore diversity) to an area where only waterfowl are present (low herbivore diversity) would represent a contrast where one herbivore group, large herbivores, is removed.

**Table 1:** Herbivore diversity contrasts considered in the systematic review, based on functional groups of herbivores (F1, F2, F3, and invertebrates [inv])

Numerical change	Identity of change	Herbivore diversity contrast	N of records
0	No contrast	F1, F2, F3 | F1, F2, F3	1(23)
		F2 | F3	1(4)
		F2, F3 | F2, F3	6(60)
		F2, F3, inv | F2, F3, inv	2(19)
		F3 | F3	2(9)
		F3, inv | F3, inv	1(2)
		inv | inv	3(16)
1	F1	F1 | zero	55(703)
		F1, F2 | F2	2(16)
	F2	F2 | zero	17(231)
	F3	F2, F3 | F2	19(643)
		F3 | zero	58(906)
		F3, inv | inv	1(4)
	inv	inv | zero	14(150)
2	F1 and F2	F1, F2 | zero	1(1)
	F1 and F3	F1, F2, F3 | F2	1(41)
		F1, F3 | zero	6(65)
	F2 and F3	F2, F3 | zero	45(793)
3	F1, F2 and F3	F1, F2, F3, inv | inv	1(27)

Functional groups were defined by [5] and represent: F1 limnic-habitat associated herbivores, migrating outside the Arctic for winter, with undifferentiated guts and feeding mainly on graminoids (waterfowl; paragon *Anser anser*); F2 immobile, burrowing species with hindgut fermenting digestive physiology (paragon *Synaptomys borealis*); and F3 large-bodied facultative-generalist species for which shrubs and lichens are an important diet component (paragon *Lepus timidus*). Numerical change indicates the difference in groups between high and low diversity areas reported in each study. Identity of change describes which group of herbivores differed between high and low diversity areas. Herbivore diversity contrast specifies the groups of herbivores present in high and low diversity areas (high | low). Number of records indicates how many articles and studies (in brackets) reported each type of contrast. Note that some articles and studies had “no contrast” even if they passed the eligibility criterion for comparator; studies with “no contrast” were not considered in further analyses

### Reasons for heterogeneity and selection of potential effect modifiers

Key sources of heterogeneity were identified based on expert knowledge and discussions with relevant stakeholders in the protocol development team [24]. The list of variables identified as potential effect modifiers was presented in Table 1 in [24] and was included as part of the coding template. For outcome variables reported by at least 10 articles, we considered the following sources of heterogeneity:

- Comparator: the comparator was quantified as herbivore contrasts (i.e., the difference between the high and low herbivore diversity levels), in terms of the number of herbivore groups or the identity of those groups.
- Study length: the length of the study (in years) was computed as the difference between the end and the start year of the study. This variable was considered because the length of herbivore exclusion is known to affect ecosystem responses [43].
- Context: variables describing the environmental context (hereafter ‘ecological modifiers’) were either coded during data extraction or extracted from existing data layers (see Table S4.8 in Additional file 4). Data availability for these variables differed, so only some of them were considered in the models (see Data synthesis).
- Other study information: to assess potential sources of publication bias, we included effective sample size (small study effect) and publication year of the study (decline effect) as recommended by [27]. Overall risk of bias (see Study validity assessment) was also considered as a source of heterogeneity. Variables relating to study type, e.g., spatial extent and resolution, were also considered as a potential source of scale dependency. In addition, ways of estimating standard deviation when calculating effect sizes rely on several assumptions (see Data synthesis and presentation), thus the method for estimating errors was also included as a potential modifier.

### Data synthesis and presentation

An overview of the studies included in the systematic review is provided as a narrative synthesis. In addition, a quantitative synthesis (meta-analysis) was conducted to assess the effects of herbivore diversity on tundra ecosystems for outcome variables reported by enough articles. All analyses were conducted in R 4.2.1 [44]. Code for conducting the analyses can be found here: https://github.com/JamesDMSpeed/ArcticHerbivoreDiversitySystematicRev.

Estimates of effect sizes of the outcome variables were calculated as the standardized mean difference (Hedges’ g) using the “escalc” function of the *metafor* package [45] based on means, SD and sample size for two groups (high versus low herbivore diversity). For each study *i* Hedges’ g $({d}_{i})$ was calculated as:

$${d}_{i}= \frac{({\overline{x} }_{iH}-{\overline{x} }_{iL})}{{s}_{i}}$$

where ${\overline{x} }_{iH}$ and ${\overline{x} }_{iL}$ were the means for the values of the outcome variable in the high and low herbivore diversity groups, and ${s}_{i}$ is the pooled standard deviation, calculated as:

$${s}_{i} =\sqrt{\frac{{(n}_{iH}-1){SD}_{iH}^{2} +{ (n}_{iL}-1){SD}_{iL}^{2}}{{n}_{iH}+{n}_{iL}-2}}$$

where ${n}_{iH}$ and ${n}_{iL}$ were the sample sizes of the high and low herbivore diversity, and ${SD}_{i}$ was the standard deviation. Variance for Hedges’ g was calculated as:

$${var}_{i}= \frac{{n}_{iH}+ {n}_{iL}}{{n}_{iH}{n}_{iL}}+ \frac{{d}_{i}^{2}}{2({n}_{iH}+ {n}_{iL})}$$

For studies reporting standard errors [2109 studies] we calculated the standard deviation as:

$${SD}_{i}= {SE}_{i}\times \sqrt{n}$$

For studies reporting 95% confidence intervals (178 studies), we estimated the standard deviation by dividing the length of the confidence interval by the corresponding value from a t-distribution with n–1 degrees of freedom, and then multiplying by the square root of the sample size [46]. For studies reporting medians and interquartile ranges (IQR; 255 studies), we used the package *estmeansd* [47] to estimate means and standard deviations. For studies reporting ranges (11 studies), SD was estimated as $\frac{1}{4}({max}_{i}-{min}_{i}$) for each herbivore diversity group.

When studies did not report any measurement of variability (448 studies), we imputed missing values using the function “impute_SD” in the package *metagear* [48] as recommended by [27], applied to each outcome variable. This function fills in missing values of SD based on resampling techniques using the coefficient of variation of available complete records (i.e., studies reporting the same outcome variable that did report SD) [48]. In some cases, the imputed values led to extreme effect size values; we therefore truncated our dataset to exclude 0.5% of the values at the extreme ends of the distribution (n = 36 studies). We re-ran the analyses not removing these outliers, and results were largely consistent (Figure S4.2 and Table S4.7 in Additional file 4). In addition, some ways of estimating standard deviations presented above rely on different assumptions. For example, estimates based on IQR work best when data are normally distributed, but often articles report medians and IQRs when data are not normally distributed. In our analyses, we controlled for the potential effect of these assumptions by including the method of estimating error as a moderator (see Reasons for heterogeneity and selection of potential effect modifiers).

For studies reporting results of a one-way ANOVA (7 studies) we used the function “esc_f” in the *metafor* package to calculate Hedges’ g. For studies presenting results of a t-test (9 studies) or effect sizes reported as raw comparison of means (1 study) we used the function “escalc”. Other types of effect sizes (e.g., response ratios or relative differences in means) could not be transformed to Hedges’ g and had to be excluded from quantitative synthesis (171 studies). When studies reported several types of outcome data (e.g., mean and variance of the outcome variable and a value for a statistical test), the mean and associated variability data were prioritized.

We used multi-level meta-analytic models [27] to account for the potential non-independence among effect sizes arising from studies reported by the same article by including article ID and study ID as nested random factors. In addition, we accounted for non-independence among sampling variances with a variance–covariance matrix. In our dataset, such non-independence could arise from studies reporting measurements of different proxies for the same response variable (e.g., articles reporting plant N content and plant P content, which are both a proxy for plant quality), or from studies reporting measurements from a shared control, as in the case of size-selective exclosure studies where pairwise comparisons between experimental treatments were extracted as separate studies. Test statistics were calculated assuming a t-distribution with adjusted degrees of freedom, as recommended by [27] for model inference in meta-analyses with a small number of articles.

We calculated intercept-only models for outcome variables measured by at least five articles (i.e., overall quantitative synthesis), to assess whether a change in herbivore diversity significantly affected the outcome variable. In most studies the contrast in herbivore diversity was provided by the complete exclusion of herbivores (i.e., the low diversity area had zero herbivores; 2,337 studies, 82.1%). Therefore, the interpretation of the effect of herbivore diversity could be confounded by the effect of completely excluding herbivores. To check if this was the case, we re-ran the intercept-only models removing studies where the low level of herbivore diversity was zero (i.e., including only partial exclusion studies; Figure S4.4 in Additional file 4).

For outcome variables reported in at least 10 articles, we built models including herbivore contrast (either numerical change in groups of herbivores or the identity of the herbivore groups) as a moderator (i.e., meta-regression models). In addition, we included the type of study (complete or partial exclusion study) as a moderator to disentangle the effect of herbivore diversity and herbivore exclusion. For these outcome variables, we also constructed models including the effect of potential ecological modifiers and other sources of heterogeneity (see Reasons for heterogeneity and selection of potential effect modifiers).

Publication bias was checked to ensure the validity of meta-analytic inferences, by assessing two aspects. First, we checked for *small study effect* where small-sized studies, with high uncertainty due to their low replication can have stronger treatment effects than larger studies. Second, we assessed the *decline effect* (or time-lag bias) where older studies report an effect but more recent studies may not, as significant results tend to be published first [27]. Small study effect was tested by including the uncertainty of effect size, calculated based on the effective sample size as a moderator in the models [27]. Decline effects were tested by including publication year (centered) as a moderator in the meta-regression models.

Some of the ecological modifiers considered as potential moderators of the effect of herbivore diversity were strongly correlated and/or had many missing values (Table S4.8 in Additional file 4), and thus were not considered further in the analyses. Ultimately, the ecological modifiers included in our models were: elevation, geodesic distance to treeline and to the coast, mean summer temperature (Jun–Aug), mean annual precipitation, recent warming (change in mean temperature from 1951–1980 to 2000–2020), recent greening (extent of change in cumulative daily growing season NDVI in 1982–2014), soil type, and extent of permafrost. Details on data sources, calculations and units for ecological modifiers are included in Additional file 4.

For each outcome variable we ran meta-regression multi-level meta-analytic models including one moderator at a time and assessed their effect by comparing the model to the intercept-only model. When several moderators had a significant effect on an outcome variable, we built multi-moderator meta-regression models (Additional file 4). We assessed model fit by comparing these models to the corresponding intercept-only models with a Log-likelihood Ratio test. We checked the robustness of our results to the presence of influential studies (sensitivity analyses) calculating Cook’s distance using the ‘leave-one-out’ approach recommended by [27]. Cook’s distance calculates the Mahalanobis distance between the predicted effect based on the full dataset and the predicted effect based on the dataset excluding one study at a time. We considered values of Cook’s distance > 1 to be influential studies [49] and re-ran the analyses without influential studies (only one study; Additional file 4). Finally, the dataset and some visualization tools are included in an interactive map server for ease of additional exploration (Additional file 5).

## Review findings

### Review descriptive statistics

**Fig. 2:**
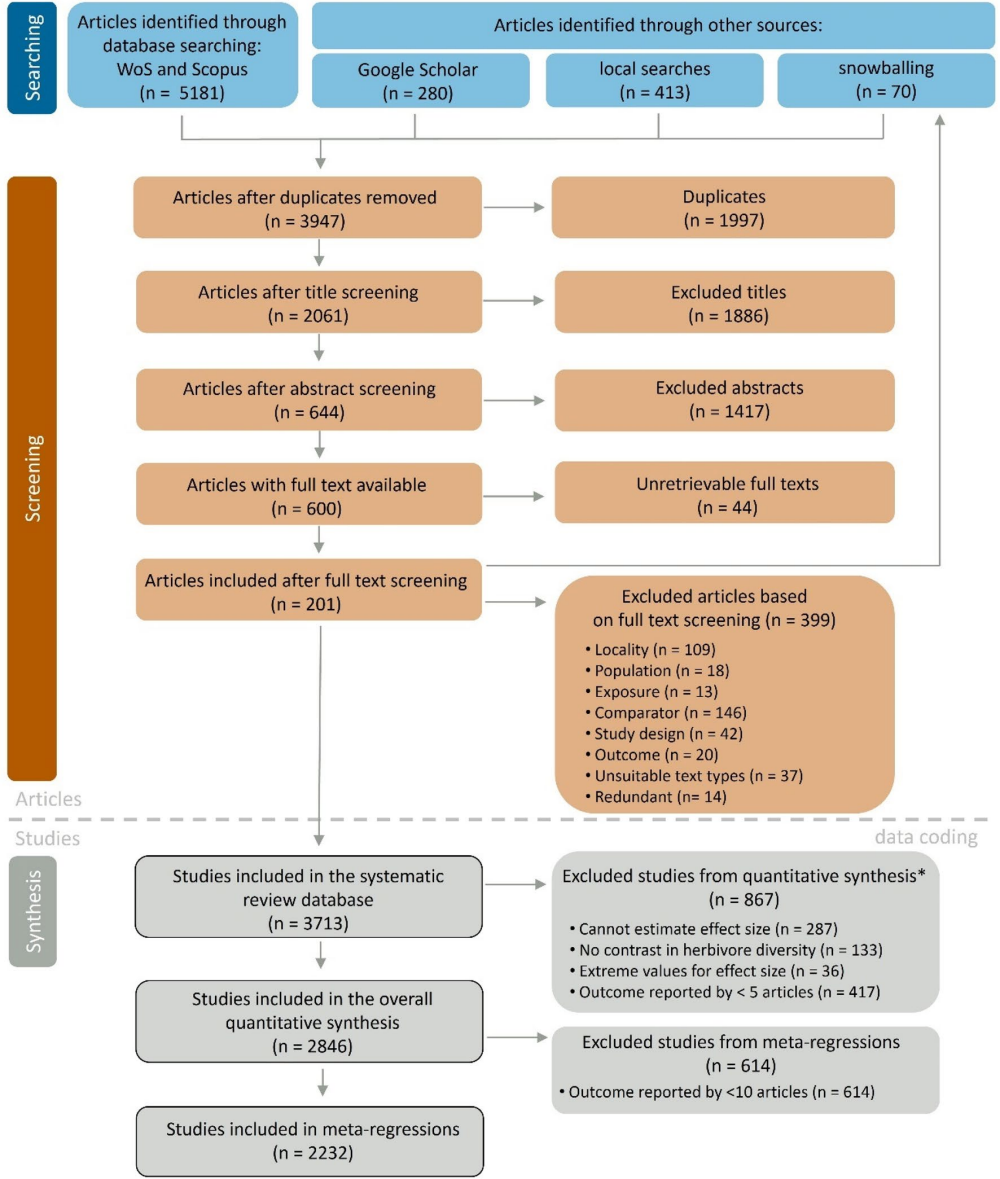
RepOrting standards for Systematic Evidence Syntheses (ROSES) diagram, outlining the process of searching, screening and synthesis for the systematic review. The diagram indicates the inclusion and exclusion process and the numbers of studies retained or excluded at each stage. The ROSES diagram is based on [30]. *Criteria for excluding studies from the quantitative synthesis are non-exclusive (the total sum is less than the numbers reported for each category separately)

We retrieved 5181 articles from Web of Science and Scopus, 280 from Google Scholar, 413 from searches in local and regional databases, and 70 articles through snowballing (Fig. 2). After removing duplicates (1997 records) and screening for eligibility at title, abstract, and full-text stages, the final database included 201 articles, representing 3713 studies. Articles reported between 1 and 171 studies (mean ± SE = 18.5 ± 1.91 studies). The full list of articles, including reasons for exclusion, is included in Additional file 2.

**Fig. 3:**
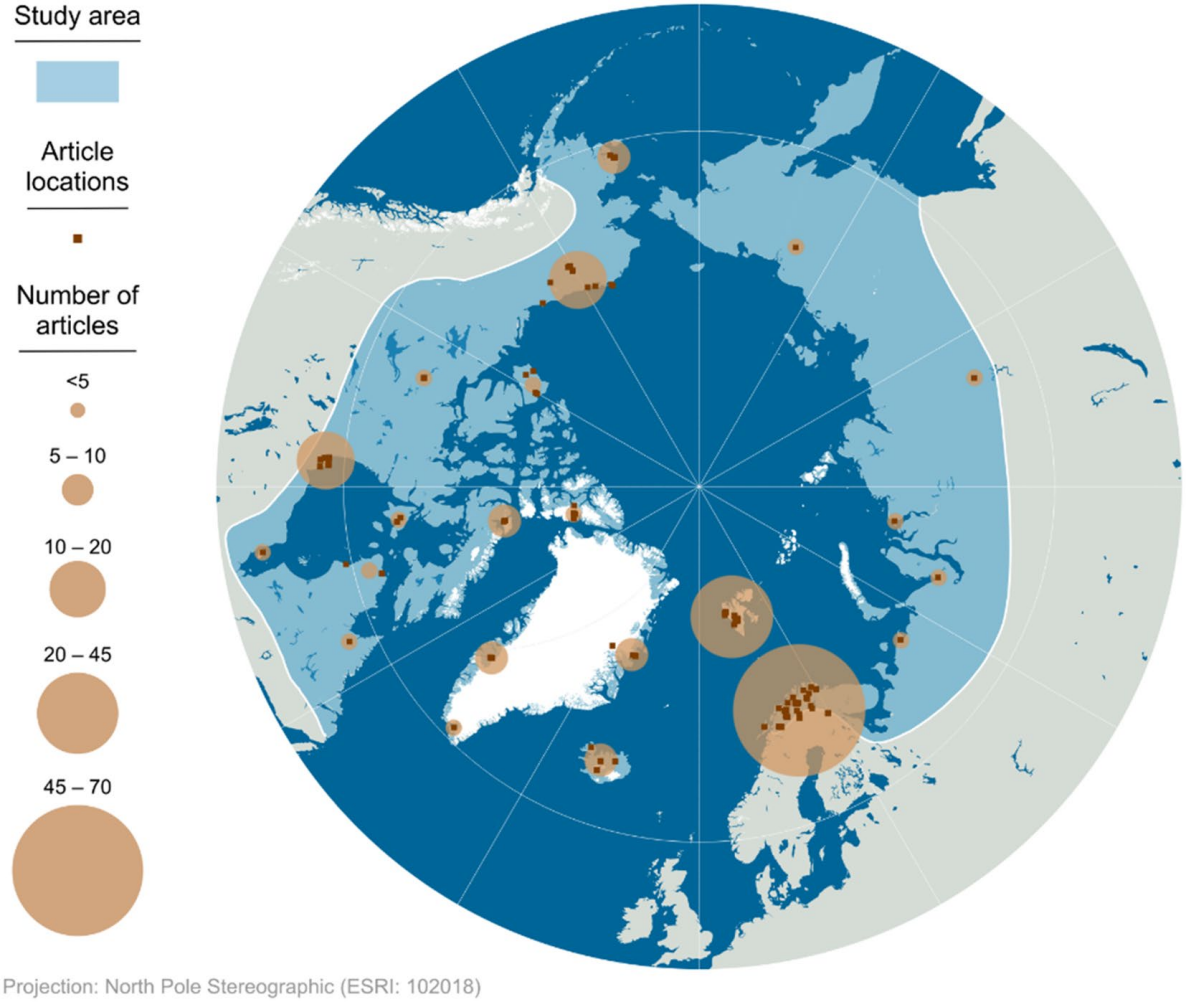
Location of the articles included in the systematic review across the Arctic were clustered around well-established research sites. Multi-study sites with coordinate centroids not on land were excluded from this graphical representation (n = 6). The study area is indicated in light blue. Dots indicate article locations and sizes of the circles indicate clusters of articles

Except for one article in Finnish (including 124 studies), all included articles were in English and published between 1982 and 2021. Less than half of the included articles (70 articles, 34.8%) had a management focus, and only 13 articles (6.5%) mentioned conservation issues. Articles clustered in mainland Norway, Sweden, and Finland (70 articles, 35.9%; Fig. 3) and Svalbard (24 articles, 12.3%), with other article clusters found on Hudson Bay (19 articles, 9.7%) and the northern coast of Alaska (18 articles, 9.2%).

Reporting of the occurrence of herbivores at the sites was often based on references to other published studies or to wildlife monitoring programmes (70 articles, 34.8%), or was mentioned by the authors without supporting references (81 articles, 40.3%). A total of 38 articles (18.9%) quantified the occurrence of herbivores at their sites, while in 13 articles (6.4%), the source of information for the occurrence of herbivores at the sites was unclear. There was also a general lack of reporting of ecological context variables referring to soil; soil chemistry, soil texture and soil moisture were only reported in 14.4%, 8.5% and 36.3% of the articles, respectively. Whether permafrost occurred or not at the study sites was reported by 22.9% of the articles.

### Narrative synthesis including study validity assessment

#### Outcome variables

**Fig. 4:**
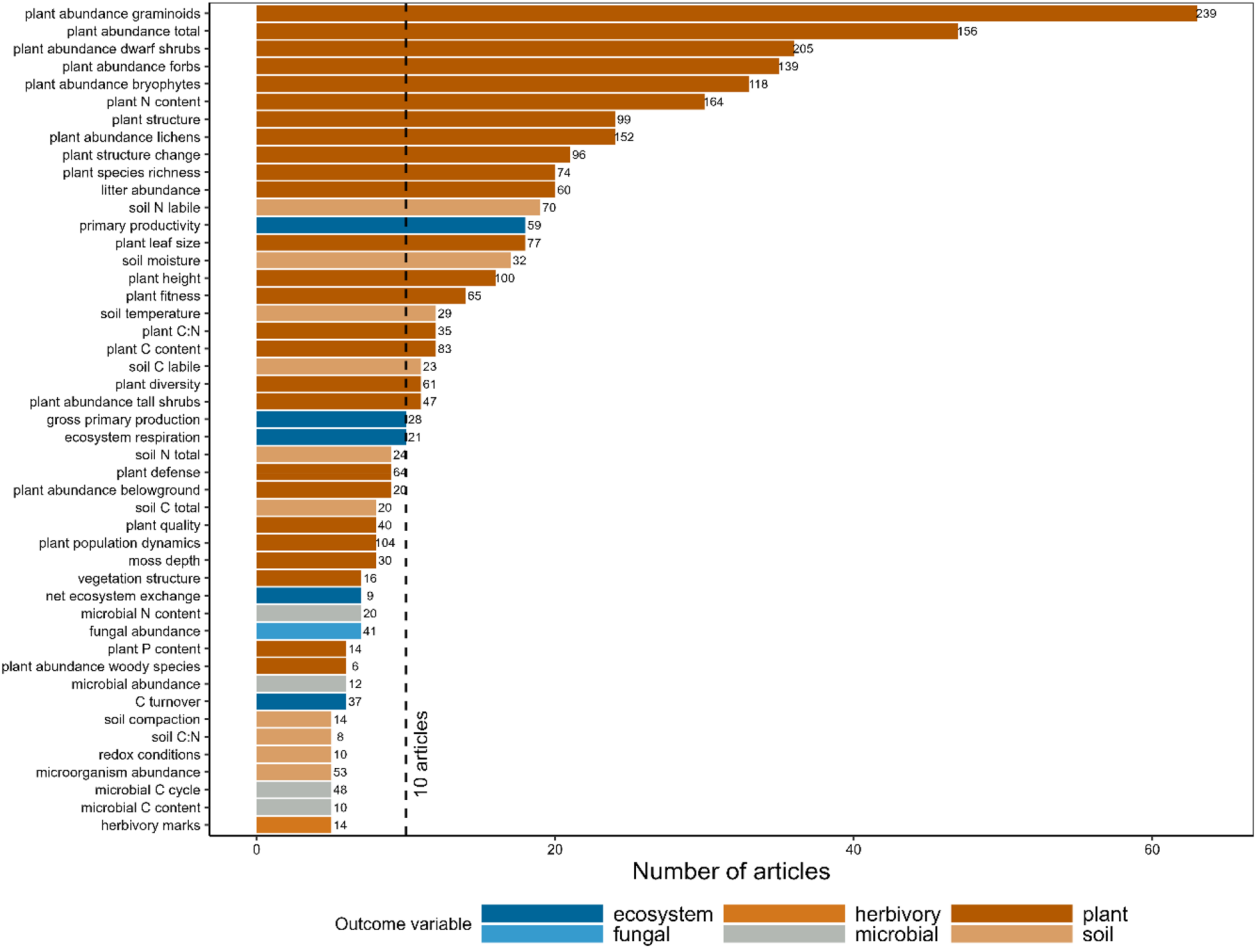
Most of the outcome variables used in quantitative synthesis in the systematic review (i.e., reported by at least five articles) referred to plants. The 47 outcome variables are ordered by the number of articles reporting each variable; number of studies is indicated to the right of the bars. The vertical dashed line denotes 10 articles, the threshold for including outcome variables in meta-regressions. Colours indicate broader classes of variables. The full list of outcome variables is presented in Additional file 4

The studies included in the systematic review reported 321 outcome variables reflecting different ecosystem processes, functions, and properties of terrestrial ecosystems in the Arctic, which were grouped into 101 broader groups for further synthesis (Table S4.2 in Additional file 4). A total of 47 outcome variables were reported by at least five articles, and 25 by at least 10 articles (Fig. 4). The most studied groups of outcome variables were graminoid abundance (63 articles, 239 studies), total plant abundance (47 articles, 156 studies), dwarf shrub abundance (36 articles, 205 studies) and forb abundance (36 articles, 139 studies). Twenty-three outcome variables were reported only by one article and included outcomes like plant disease or the species richness of plant-dwelling invertebrates. The full list of outcome variables, including the original variables reported by the studies, is presented in Additional file 3.

#### Herbivores and herbivory diversity contrasts

The taxonomic resolution at which herbivore assemblages were reported, from species or subspecies to broader groups like ‘large herbivores’ or ‘eriophyid mites’ (Table S4.3 in Additional file 4), differed between studies. Some studies provided a detailed account of all herbivores known to occur in the study area, whereas others reported only the most abundant herbivores. Further, reporting of herbivore identity differed even between studies conducted at the same locality.

According to our definition of herbivore diversity contrast as the difference between the high and low herbivore diversity areas, some studies had no contrast because they compared areas with different assemblages of herbivores belonging to the same functional groups, or because high and low diversity areas included the same number of herbivore groups (‘no contrast’ category in Table 1; such studies were not considered in further analyses). Contrasts in herbivore diversity including invertebrate herbivores were only reported by 14 articles (150 studies) referring to the exclusion of invertebrate herbivores (i.e., invertebrate herbivores vs zero). Other studies mentioning invertebrate herbivores reported them as present in both high and low diversity areas, and thus invertebrate herbivores did not contribute to the contrast in diversity. The most common contrasts in herbivore diversity were exclusion studies of large-bodied herbivores (F3 versus zero; 58 articles, 906 studies), waterfowl (F1 versus zero; 55 articles, 703 studies), or large-bodied and smaller resident herbivores (F2 and F3 versus zero; 45 articles, 793 studies, Table 1), followed by removal of large-bodied herbivores where smaller resident herbivores were present (F2 and F3 versus F2; 19 articles, 643 studies).

Most studies were experimental (85.5%, 3175 studies) and most of them used exclosures to create the contrast in herbivore diversity (2516 studies), while others simulated herbivory (444 studies) or used enclosures where a known number of animals is kept within a fixed area (138 studies). Among the studies using exclosures, 636 studies belonging to 15 articles specifically addressed the effect of different groups of herbivores using size-selective exclosures, where different groups of herbivores are excluded sequentially depending on their body size (Table S4.9 in Additional file 4).

#### Study validity assessment

Of the 3713 studies included in the systematic review, 5.6% had an overall low risk of bias, 67.5% had an overall medium risk of bias and 26.9% had an overall high risk of bias (Figure S4.5 in Additional file 4). Among the assessed criteria, confounding bias (criterion 1) had the highest number of studies with a high risk of bias (826 studies).

#### Inclusion of studies in quantitative synthesis

From the 3713 studies included in the systematic review database, 867 studies could not be included in quantitative synthesis (Fig. 2). For 287 studies, it was not possible to calculate effect sizes because they: (i) reported variability of zero, sample sizes of one or lacked variability measurements that could not be imputed (114 studies), or (ii) they reported results that could not be transformed to Hedges’ g (other types of effect sizes: 171 studies, or results of a paired t-test: 1 study). We excluded 36 studies where the imputation of missing values led to extreme outliers for effect size estimates (see Additional file 4 for analyses without removing extreme values). Further, 133 studies could not be included in the quantitative synthesis because they did not present an herbivore diversity contrast (Table 1), and 417 studies had to be excluded because they reported on outcome variables measured in less than five articles. Therefore, 2,846 studies were finally included in the overall quantitative synthesis (intercept-only models) of which 2232 studies were included in meta-regressions.

### Data synthesis

#### Overall effect of herbivore diversity

**Fig. 5:**
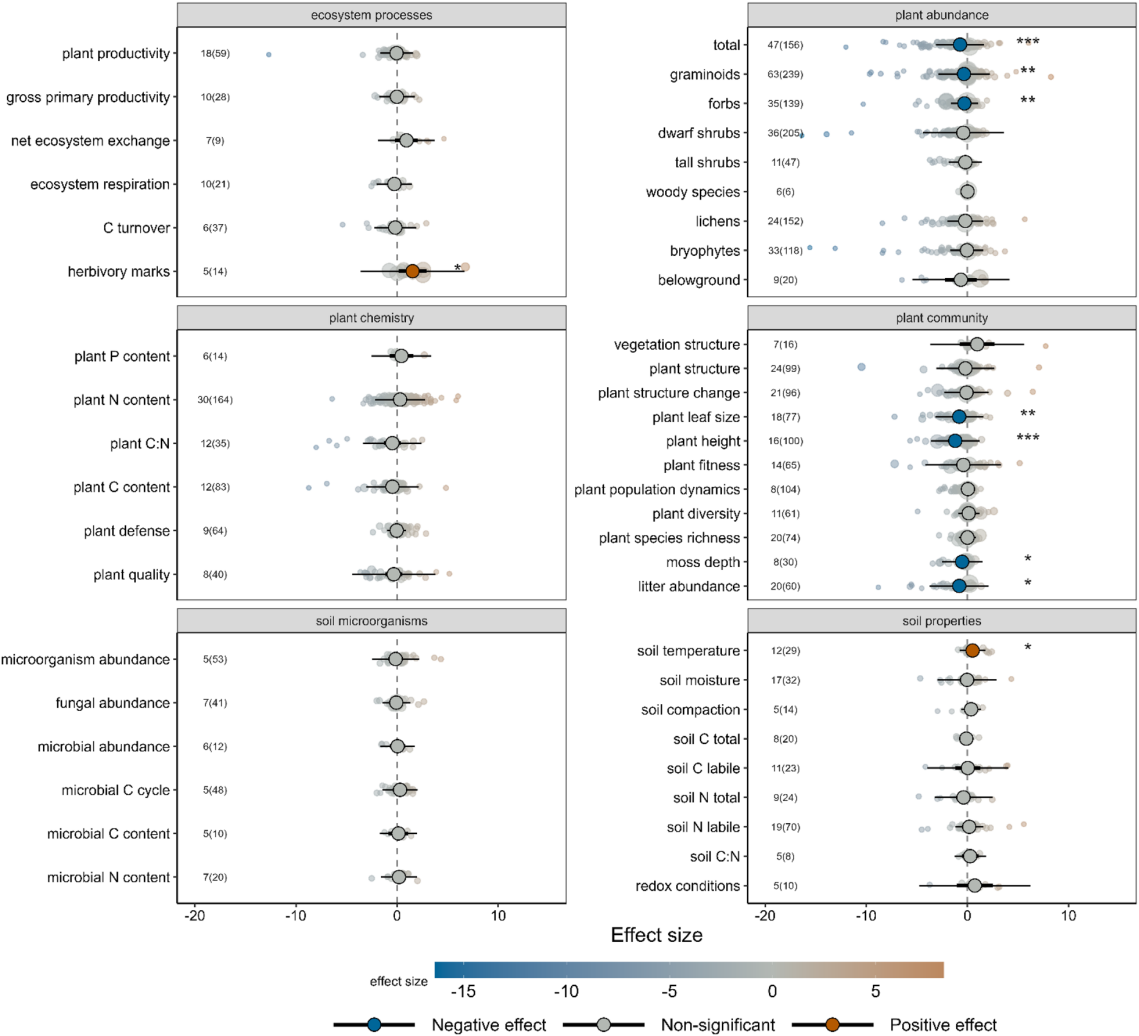
Herbivore diversity significantly affected nine outcome variables, mainly related to plant community and plant abundance, as assessed using intercept only models on the 47 outcome variables reported by at least five articles. Number of articles and studies (in brackets) are indicated for each outcome variable in small font to the left of the graphs. Circles represent overall effect sizes with colour indicating the magnitude of the effect. Thin lines represent prediction intervals and thicker lines (often hidden behind the overall effect size) represent confidence intervals. Significance of the effect is indicated with darker colours and asterisks (*p < 0.05; **p < 0.01; ***p < 0.001)

The intercept-only models built for the 47 outcome variables reported by at least five articles revealed significant effects of the contrast in herbivore diversity (i.e., high versus low herbivore diversity) on nine outcome variables (Fig. 5). Greater herbivore diversity led to increased abundance of herbivory marks (e.g., signs of feeding) and soil temperature, and to reduced total abundance of plants, graminoids and forbs, plant leaf size, plant height, moss depth, and litter abundance. After removing the complete exclusion studies (where the low level of herbivore diversity was zero) from the analyses, intercept-only models could only be run for five outcome variables. In these models, the effects of herbivore diversity remained non-significant or became non-significant, compared to the models including all studies (Figure S4.4 in Additional file 4). The analyses without exclusion studies are based on a much smaller sample size and could only be run for a subset of outcome variables but could be an indication that the effects of herbivore diversity may not be clearly distinguishable from the effects of herbivore exclusion.

#### Moderators of the effect of herbivore diversity

**Fig. 6:**
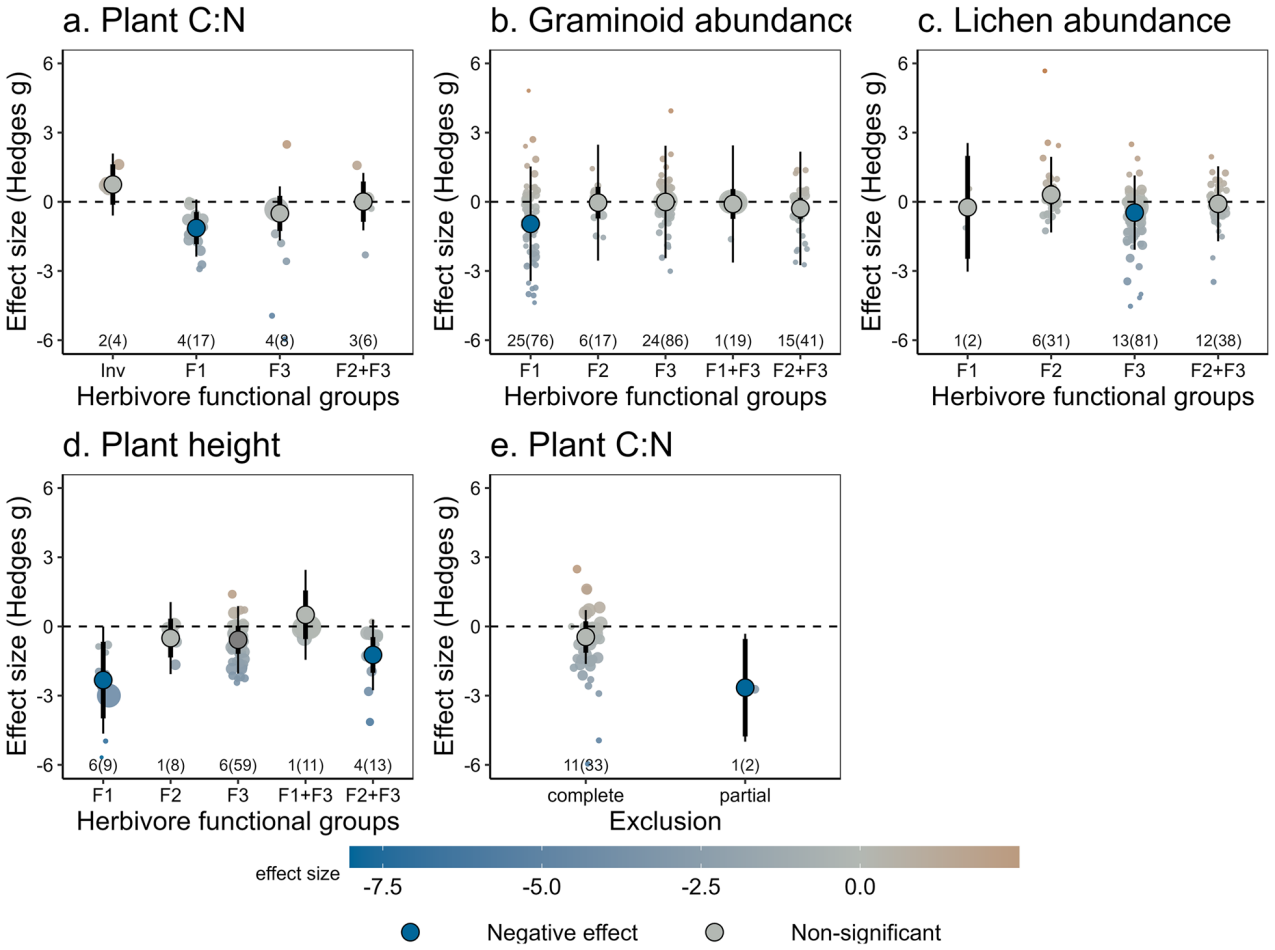
The impact of herbivore diversity on plant C:N ratio, graminoid and lichen abundance, and plant height was modulated by the identity of herbivore functional groups (**a**–**d**) or the partial exclusion of herbivores (**e**). Functional groups of herbivores were defined by [5] and represent: F1 limnic-habitat associated herbivores, migrating outside the Arctic in winter, with undifferentiated guts and feeding mainly on graminoids (waterfowl; paragon *Anser anser*); F2 immobile, burrowing species with hindgut fermenting digestive physiology (paragon *Synaptomys borealis*); F3 large-bodied facultative-generalist species for which shrubs and lichens are an important diet component (paragon *Lepus timidus*); and Inv for invertebrates. Exclusion refers to studies where all herbivores (complete exclusion) or only some groups of herbivores (partial exclusion) are removed. Circles represent overall effect sizes with colour indicating the magnitude of the effect. Thin lines represent prediction intervals and thicker lines (often hidden behind the overall effect size) represent confidence intervals. Significance of the effect, as per the meta-regression models, is indicated with darker colours. The number of articles and studies (in brackets) are indicated in small font; note the small sample sizes for some levels of variables

The identity of herbivore functional groups and the partial exclusion of herbivores modulated the effect of herbivore diversity on plant C:N ratio (i.e., a measure of plant quality, considered to be of high quality when the ratio is low), graminoid and lichen abundance, and plant height (Fig. 6). The presence of waterfowl (F1) reduced plant C:N ratios (Fig. 6a). Herbivore diversity significantly reduced plant C:N ratio in partial exclusion studies (i.e., those that did not exclude all herbivores) but this effect was driven by the only article (including two studies) that measured plant C:N ratio and did not completely exclude all herbivores (Fig. 6e). The abundance of graminoids was negatively affected by waterfowl, but this effect was not detected when both waterfowl and large-bodied herbivores (F1 and F3) were present, suggesting a compensatory effect between these two groups of herbivores (Fig. 6b; Fig. 1). A similar pattern was found for lichen abundance, which declined when large-bodied herbivores (F3) were present, but not in the presence of both large and smaller herbivores (F2 and F3; Fig. 6c). Similarly, for plant height the negative effect of waterfowl (F1) was not detected when both waterfowl and large-bodied herbivores (F1 and F3) were present together. Conversely, large-bodied herbivores (F3) only reduced plant height when occurring together with smaller herbivores (F2) but not when occurring separately, suggesting an additive effect of these groups of herbivores (Fig. 6d).

**Fig. 7:**
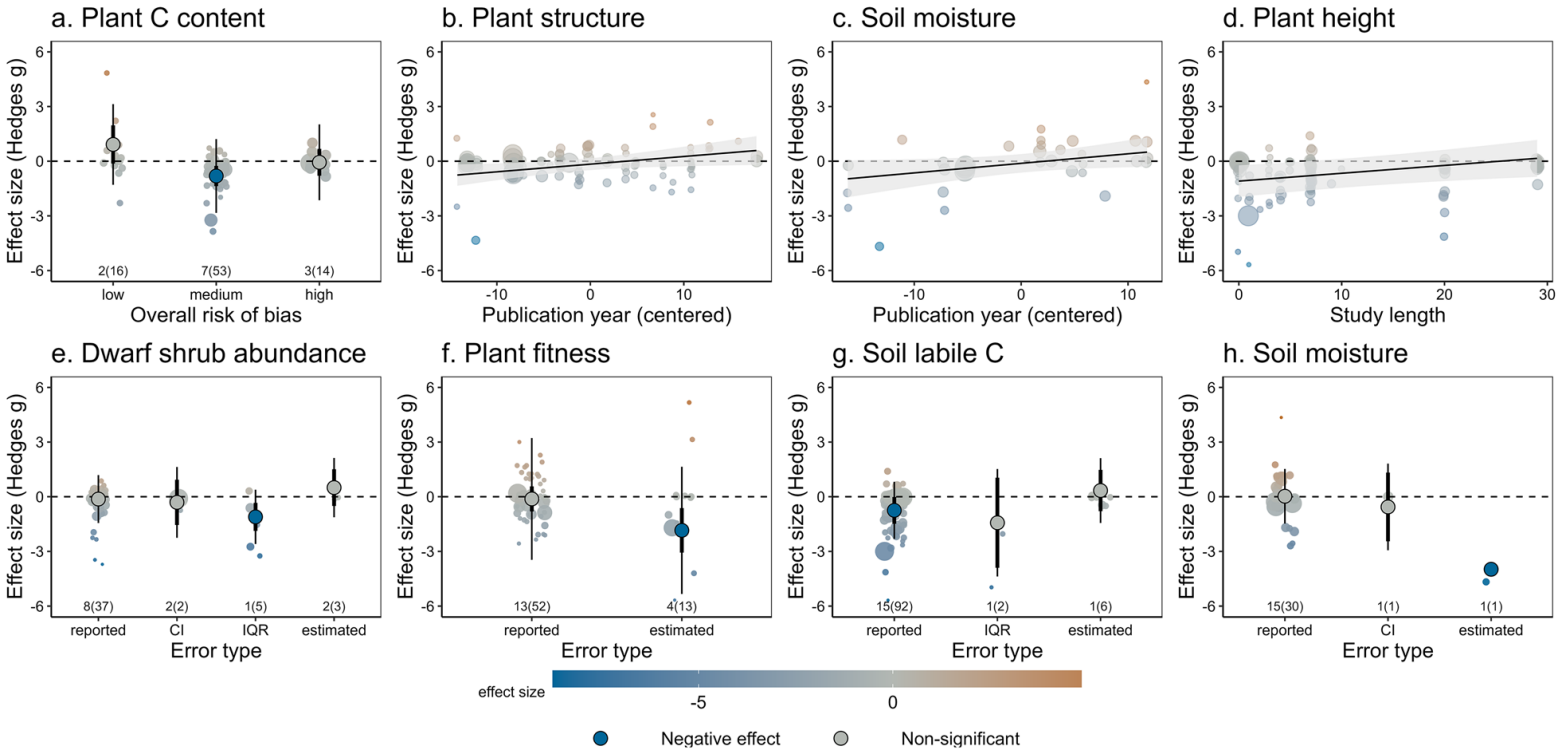
The effect of herbivore diversity on plant C content, plant structure, soil moisture, plant height, dwarf shrub abundance, plant fitness and soil labile C was modulated by study validity (**a**), publication bias (decline effect; **b**, **c**), length of the study (**d**) and type of error estimate (**e**–**h**). For categorical moderators, circles represent overall effect sizes with colour indicating the magnitude of the effect, thin lines represent prediction intervals and thicker lines (often hidden behind the overall effect size) represent confidence intervals. Significance of the effect, as per the meta-regression models, is indicated with darker colours. The number of articles and studies (in brackets) are indicated in small font; note the small sample sizes for some levels of variables. For continuous moderators, lines represent model fit with 95% confidence intervals

Study validity moderated the effect of herbivore diversity on plant C content, with herbivore diversity only reducing plant C content in studies that were scored as having an overall medium risk of bias, but not for those with low or high risk of bias (Fig. 7a). However, this effect was driven by the presence of an influential study (Figure S4.6 in Additional file 4) with a strong positive effect size and low risk of bias; when this study was removed from the analyses, the effect of the overall risk of bias became non-significant. We found evidence of publication bias for plant structure and soil moisture (Fig. 7b, c), where older studies tended to report significantly negative effects of herbivore diversity and more recent studies tended to report positive effects.

**Fig. 8:**
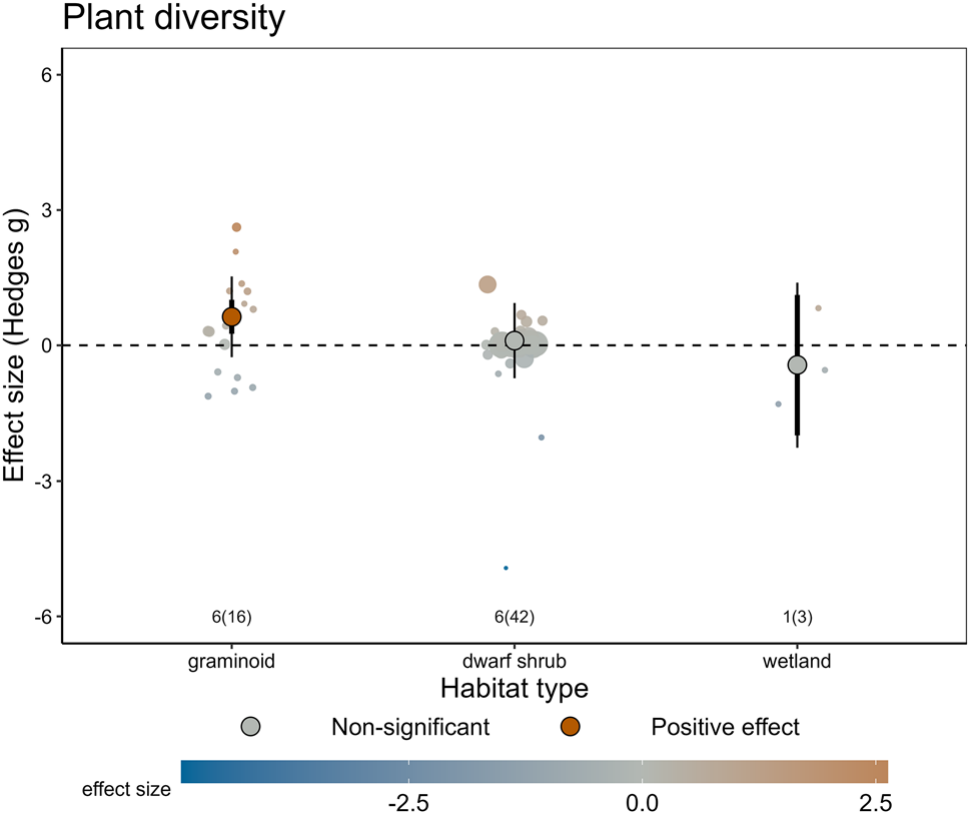
The effect of herbivore diversity on plant diversity was modulated by habitat type. Circles represent overall effect sizes with colour indicating the magnitude of the effect, thin lines represent prediction intervals and thicker lines (often hidden behind the overall effect size) represent confidence intervals. Significance of the effect, as per the meta-regression models, is indicated with darker colours. The number of articles and studies (in brackets) are indicated in small font; note the small sample sizes for some levels of variables

Other methodological aspects that moderated the effect of herbivore diversity were the length of the study, and the method used for estimating standard deviations when calculating effect sizes (e.g., whether SDs were reported directly by the studies, or estimated based on CIs, IQRs or by imputation; Fig. 7e, h). The length of the study affected the responses of plant height (Fig. 7d), where the negative effect of herbivore diversity became non-significant in longer-term studies. Importantly, we accounted for the effects of these methodological aspects in our multi-moderator meta-regression models (Figs. 6, 7, 8) and corrected the estimates of the effects of ecological moderators by including moderators related to study validity, publication bias and scale dependence as model covariates. These models significantly improved model fit, relative to the corresponding intercept-only models (Table S4.10 in Additional file 4).

The only ecological moderators that modulated the effects of herbivore diversity was habitat type. Greater herbivore diversity increased plant diversity in graminoid tundra but not in other habitat types (Fig. 8).

### Review limitations

The systematic review used a search string developed for a previously published systematic map on the effects of herbivory on Arctic vegetation [12]. The search string focused on the region/system (terrestrial Arctic ecosystems) and exposure (herbivory) and did not pose any restriction to the outcome or comparator. Therefore, the search string was deemed broad enough to include all articles relevant to the systematic review [24] at the expense of reducing the proportion of relevant studies retrieved by the search, that is, its specificity. Hence, considerable screening work was required. Indeed, 83% of articles (3303 out of 3947 articles, after excluding duplicates) were removed after the title and abstract screening stages. However, we were surprised by the number of relevant articles retrieved through snowballing (70 articles, 14 of which were included in our database after full text screening). In most cases, these articles may not have been identified by our search string because the title and abstract did not include any reference to Arctic, subarctic or tundra. This was, for example, the case for many goose grazing studies conducted in coastal areas (e.g., [50, 51]) or for studies targeting Arctic herbivore species like reindeer or muskoxen but not mentioning Arctic, subarctic or tundra specifically (e.g., [52, 53]). A more inclusive search, including synonyms or adding key species names of Arctic herbivores could have improved this situation by retrieving articles that are now missing, but it is still unlikely that we would have retrieved articles from habitats not described as Arctic or tundra. In any case, this demonstrates the value of additional bibliographic search efforts like snowballing in identifying potentially relevant studies that were not captured by the search string.

One of the main limitations of this systematic review was that most articles included were not specifically designed to address questions related to herbivore diversity. We included studies that removed all herbivores (i.e., complete exclusion studies) deliberately because such studies also represent a change in herbivore diversity. For example, exclusion studies can represent different contrasts of herbivore diversity if they exclude different numbers of functional groups of herbivores. However, including these studies also means that our results can be confounded by the presence/absence of herbivores rather than by a change in herbivore diversity per se. It is also important to note that the assessment of complete exclusion is based on the herbivores reported by the studies, which often do not consider invertebrate herbivores, birds, or small mammals. Thus, what we consider here complete exclusion studies refers in most cases only to vertebrate herbivore exclusion. Few studies experimentally manipulated herbivore diversity (15 articles including 636 studies) using size selective exclosures (Table S4.9 in Additional file 4). As any experimental manipulation, size-selective exclosures have limitations and are expensive to maintain, but they provide invaluable information on the role of different herbivore assemblages in ecosystem functioning [54].

A clear challenge when synthesizing the evidence was the quality of reporting by different articles. The level of taxonomic resolution differed among studies, with some reporting subspecies and others broadly grouping herbivores into larger categories. Further, reporting of herbivores was often incomplete, with some studies reporting all herbivore species known to occur in the study area while others only mentioned the more frequent herbivores. Our analyses are based on occurrence of herbivores at the study areas and include the richness of groups of herbivores as a proxy for diversity. Including diversity at the species-level or some estimates of the densities of herbivores would have provided more nuanced insights into the effects of herbivore diversity, but such density estimates are not available globally (but see [55]) or even at a local scale [56]. For instance, we found that the direct quantification of herbivore abundance was relatively rare (19% or 38 articles), although herbivore densities can drive the effect of herbivore diversity [57]. Furthermore, herbivore abundance may be even more important when addressing the effect of herbivore diversity on Arctic ecosystems, which are characterized by strong cyclic population dynamics [58, 59].

One final point worth mentioning concerns the consistency of assessment of the risk of bias between reviewers. Although reviewers largely agreed in the overall assessment, assessment of individual risk criteria was less consistent. During protocol development, we created decision trees for each of the seven bias criteria identified by [37], adapted to our study question, to make this process as transparent and straightforward as possible. Even when using the decision trees, reviewers sometimes disagreed in their assessment of individual bias criteria, perhaps due to the subjective nature of the relative importance of different parts of the decision tree. For example, some reviewers took ambiguity in the randomness of initial site selection as cause for concern regarding the risk of post-intervention sampling bias (criterion 2), whilst others saw the random allocation of treatments within-site sufficient to warrant low-risk status. Including clearer criteria, specific examples or criteria more targeted to field studies in the decision trees could have increased agreement between reviewers, but given the discrepancies outlined above we believe this would have had a minimal impact. Most of the studies included in the systematic review were classified as having a medium risk of bias, in most cases due to incomplete reporting by the studies. For instance, many studies did not clearly state if potential confounding variables had been taken into account (biases due to confounding factors, criterion 1), did not accurately describe their sampling design (risk of post-intervention sampling bias, criterion 2) or did not report whether the researcher was aware of the treatment when taking the measurements (risk of measurement bias, criterion 5). That said, some of these criteria may not be applicable to ecological field studies, where researchers collecting the data have often been involved in the design of the study and the implementation of the treatments which are usually observable to the person collecting the data, for example when taking measurements inside a fenced area. Thus, many field ecologists would not think this should be reported in their study. The development of checklists for reporting standards could help in this respect.

## Review conclusions

### Implications for policy/management

We found some evidence that the effects of different functional groups of herbivores on tundra ecosystems can either compensate for each other or cumulatively show a stronger effect. Among compensatory effects, waterfowl reduce the abundance of graminoids, but this effect is not detected when they graze together with large-bodied herbivores. Similarly, large-bodied herbivores can reduce the abundance of lichens, but when smaller herbivores are also present this effect weakens. Such effects could be attributed to altered competitive interactions between plants under selective grazing. For example, [60] argued that selective feeding of microtine rodents on vascular plants could give a competitive advantage to lichens that could partly compensate the consumption of lichens by caribou when microtines and caribou graze the same areas. Conversely, we found additive effects in the case of plant height, where small and large herbivores reduced plant height only when occurring together but not when occurring separately. These findings highlight the importance of considering the functional roles of herbivores in multi-species assemblages when trying to understand their ecosystem impacts. In systems like Arctic tundra, where herbivore functional diversity is low, the identity of herbivores—and hence the presence of specific functions in the herbivore assemblage—is likely to play an important role [54].

We found that the effects of herbivore diversity on plant diversity depended on the type of habitat, with higher herbivore diversity increasing plant diversity in graminoid tundra. Such effects have also been described in productive temperate grasslands, where assemblages composed by large and small herbivores had a positive effect on plant species richness, but small herbivores alone did not [42]. In graminoid tundra, herbivores also influence plant diversity and can dampen diversity loss associated with warming [3].

Variation in the effects of diverse herbivore assemblages on tundra vegetation communities across the tundra biome has relevant implications for management, emphasizing that there is no one-size-fits-all recommendation. Depending on the ecosystem processes (and hence the ecosystem services) that managers are trying to maximize, they will need to consider the specific effects of herbivore diversity on different environmental context, and whether those effects are additive or compensatory.

Knowledge on the combined effects of herbivores, and thus on the role of diverse herbivore assemblages in ecosystem functioning, is becoming increasingly important in the context of ongoing environmental and land use changes in the Arctic [19, 61]. Such changes can imply the loss or gain of herbivore species, both (semi)domesticated and wild. For example, grazing by *Rangifer* (both wild and semidomesticated reindeer husbandry) is increasingly threatened by land use changes [62] and Arctic greening trends have been associated with declines in migratory caribou populations [23]. In turn, trophic rewilding of herbivore communities, where more complete native large herbivore assemblages are being restored, has been proposed as a tool to mitigate some of the impacts of climate change on tundra ecosystems [13, 63]. Improving our understanding of the combined effect of different herbivores will support better decision-making in these changing contexts, where herbivore species are being lost or gained.

### Implications for research

This systematic review focused on the effects of herbivore diversity on the dynamics of tundra ecosystems. The primary question was purposely open-ended in terms of outcomes, to provide an overview of the scope and volume of research conducted on herbivore diversity in tundra ecosystems [24]. Although the studies included in the systematic review reported on 101 groups of outcome variables, most of them were related to plants, followed (far behind) by soils and ecosystem level processes. There are thus clear knowledge gaps regarding the impacts of herbivores on non-plant related outcomes.

We found that the evidence base for the effects of herbivore diversity on tundra ecosystems is also geographically and taxonomically uneven. Most of the available knowledge comes from well-established research locations. This geographic bias in tundra data has previously been described for Arctic studies [12, 64], and reflects the difficulties of conducting ecological research in remote areas. Some groups of herbivores are studied more frequently than others, with invertebrate herbivores being underrepresented and making up only 7% of articles in our compiled database. Other studies highlight the need for greater research on invertebrate herbivory in the Arctic [12, 65]. Invertebrate herbivores are an important indicator of Arctic environmental change [66], as they are found across the tundra biome and certain taxa are projected to increase in abundance and extend their distribution range with climate change [67].

Measuring herbivory in the field requires researchers to make decisions, such as selecting the study site and which species to target, as well as allocating experimental treatments to the study units. Such decisions are often also constrained by logistics, ease of access and support infrastructure and are often not clearly reported in ecological research [58]. These decisions can be prone to biases leading to overestimation of the effects being studied, ultimately compromising the reproducibility of research [68]. Some practices can be easily implemented to prevent some of these biases; for example, researcher awareness of the treatments can be avoided by blinding the identity of the treatments when samples are given random labels that do not explicitly refer to the experimental treatment, so that this knowledge does not influence the researcher when processing and analysing the samples [69]. Another simple recommendation to avoid observer bias (or at least to quantify it) is to regularly run intercalibration exercises for measurements collected by different observers. Raising awareness among researchers about conscious and unconscious biases is a key step forward [70] and together with stricter reporting standards, will enhance the reproducibility of scientific results and their usefulness for future data syntheses.

We found evidence for publication bias for two outcome variables (plant structure and soil moisture), where the year of publication modulated the effect of herbivore diversity. Older studies tended to report significantly negative effects of herbivore diversity and more recent ones tended to report positive effects. Interestingly, the direction of these changes contrasts with expectations resulting from the ‘decline effect’, where effect sizes decrease over time because significant results tend to be published faster [71]. Given that these two outcome variables were not affected by any of the ecological modifiers included in the analyses, it is unlikely that these trends are related to a shift in research focus to, for example, different habitats or different bioclimatic conditions. These trends could thus suggest a potential paradigm shift where the positive effects of herbivore diversity on ecosystem dynamics are increasingly recognised in the literature.

Despite an increasing volume of literature on impacts of herbivores in the tundra, many challenges remain to fully understand the complex impacts of herbivore diversity on Arctic systems. We found that research on the effects of herbivores on tundra ecosystems is relatively limited, but what is currently found in the literature points to the importance of the functional identity of herbivores in these low-diversity systems. There is a clear need for studies specifically measuring the effects of different herbivores, separately and in combination, on tundra ecosystems, particularly on components other than plants and with increased attention to invertebrate herbivores. While comparisons between the presence and absence of herbivores can provide a powerful experimental approach to address impacts of herbivores, we need more granularity of studies addressing a range of diversities to parse out the nuances that can emerge from different herbivore assemblages. Similarly, additional efforts should be placed on characterising the full assemblage of herbivores (both vertebrate and invertebrate) and accurately quantifying the abundance of herbivores in field studies. Our ability to synthesise information on impacts of herbivore assemblages on ecosystems would also be improved by recording key information consistently across herbivore studies to better characterise the context dependency of herbivore diversity impacts across the Arctic. Future studies should explicitly address the role of herbivore diversity to refine predictions on whether and where these shifts could mitigate or further amplify the impact of ongoing environmental and land use changes on Arctic ecosystems.

## Supplementary Information

**Additional file 1**: ROSES form for systematic review reports.

**Additional file 2**: Additional information on literature searches and full article list.

**Additional file 3**: Coded raw data from full text studies and study validity assessment.

**Additional file 4**: Extended methods and results.

**Additional file 5**: Interactive map server.

## Data Availability

The datasets supporting the conclusions of this article are included within the article and its additional files.
