# Supplementary material for: Herbivore diversity effects on Arctic tundra ecosystems: a systematic review
Source: Environ Evid. 2024 Mar 25;13:6. doi: 10.1186/s13750-024-00330-9 (PMC11378771; doi:10.1186/s13750-024-00330-9)

**Additional file 4.** Extended methods and results

# Repeatability of the screening process and critical appraisal

All articles retrieved from the searches were screened in three sequential stages: title, abstract, and full text screening. A different number of reviewers participated in each stage (**Table S4.1**): 9, 13, and 19 reviewers, respectively. The repeatability of the screening process was assessed by measuring the consistency of two independent reviewers to either include or exclude an article at each stage. A total of 3,520 titles (out of the 3,947 articles in the database after removing duplicates; 89.2%), 204 abstracts (out of the 2,050 articles included at the title stage excluding correction/replies; 10%) and 28 full texts (out of 633 articles included at abstract stage, excluding correction/replies; 4.4%) were assessed by two independent reviewers.

Thirteen reviewers extracted data from all the included articles (n = 201; **Table S4.1**) after the title, abstract and full text screening. The same reviewers also conducted the critical appraisal of the articles from which they extracted data. One reviewer (IK) independently assessed 21 of those articles that had been previously assessed by an independent reviewer.

We calculated the consistency between the assessments of the two independent reviewers using two metrics: the percent agreement and Cohen’s Kappa (κ) statistic (31). Percent agreement reflects the number of times reviewers agreed divided by the total number of screened articles, while Kappa is a measure of interrater reliability (i.e. the extent to which two or more individuals agree; (32) that incorporates chance agreement between reviewers.

**Table S4.1.** Multiple reviewers contributed to the screening of articles at the title, abstract and full text stage, and to the coding and study validity assessment. The total number of articles assessed by 2 independent reviewers at each stage is also indicated, as well as the total number of articles included in each stage.

| **Reviewer** | **Titles** | **Abstracts** | **Full texts** | **Coding and study validity assessment** |
| --- | --- | --- | --- | --- |
| LBP | 3859 | 877 | 155 | 62 |
| ICB | 3683 | 595 | 305 | 92 |
| MPB | 151 | 239 | 4 | 4 |
| BCH | 33 | 164 |  |  |
| RB | 18 |  | 12 | 2 |
| KB | 9 | 9 | 9 | 1 |
| AS | 7 | 7 |  |  |
| THMK | 7 | 7 | 9 | 5 |
| TKL | 7 | 7 | 3 | 3 |
| JSR |  | 240 | 1 | 1 |
| AJL |  | 74 | 1 |  |
| RB |  | 9 |  |  |
| OG |  | 11 |  |  |
| MdH |  | 4 |  |  |
| DSH |  |  | 70 |  |
| MGC |  |  | 30 | 17 |
| TW |  |  | 17 |  |
| JCA |  |  | 14 |  |
| IK |  |  | 11 | 28 |
| EPP |  |  | 8 |  |
| LBC |  |  | 4 | 3 |
| MD |  |  | 4 | 3 |
| MSK |  |  | 3 | 1 |
| NS |  |  | 1 |  |
| Total screened by 2 reviewers | 3520 | 204 | 28 | 21 |
| Total included at previous screening stage | 3947 | 2050 | 633 | 201 |
| Nr reviewers at each stage | 9 | 13 | 19 | 13 |

# Lists of outcome variables, plants and herbivores

## Outcome variables

**Table S4.2.** Outcome variable categories used in the systematic review, grouped into larger classes (disease, ecosystem, fungal, herbivory, invertebrate, microbial, plant and soil) and outcome variables reported by the studies. For original values of outcome variables reported by each study in the database, see outcomes in Additional file 3

| **class** | **outcome variable** | **reported outcome variable** |
| --- | --- | --- |
| disease | disease | disease frequency |
| ecosystem | acetylene reduction | acetylene reduction |
|  |  | N fixing |
|  | C turnover | C turnover |
|  |  | decomposition |
|  | CH4 flux | CH4 flux |
|  | ecosystem respiration | CO2 flux |
|  |  | ecosystem respiration |
|  | gross ecosystem exchange | gross ecosystem exchange |
|  | gross primary production | gross ecosystem photosynthesis |
|  |  | gross primary production |
|  |  | net photosynthetic capacity |
|  |  | photosynthetic efficiency |
|  | net ecosystem exchange | net ecosystem exchange |
|  | net ecosystem production | net ecosystem production |
|  | primary productivity | biomass production |
|  |  | NDVI |
|  |  | net aboveground primary productivity |
|  |  | net primary production |
|  |  | productivity |
| fungal | fungal abundance | fungal biomass |
|  |  | hyphal load |
|  |  | occurrence of dark septate endophytes |
|  |  | root fungal colonization |
|  |  | spores presence |
|  | fungal diversity | morphotype richness |
|  |  | morphotype simpson index |
| herbivory | grazing preference | grazing preference |
|  | herbivory marks | browsing frequency |
|  |  | browsing intensity |
|  |  | bud damage |
|  |  | leaf damage |
|  |  | shoot damage |
|  | herbivory rate | intake rate |
| invertebrate | invertebrate abundance | invertebrate relative abundance |
|  |  | nr individuals |
|  | invertebrate diversity | invertebrate diversity |
|  | invertebrate evenness | invertebrate evenness |
|  | invertebrate properties | relative growth rate |
|  |  | weight |
|  | invertebrate species richness | invertebrate species richness |
| microbial | fungi:bacteria ratio | fungi:bacteria ratio |
|  | microbial abundance | bacteria biomass |
|  |  | DNA |
|  |  | phospholipid fatty acids |
|  |  | RNA |
|  | microbial activity | microbial activity |
|  | microbial C content | microbial C content |
|  | microbial C cycle | alphaglucosidase activity |
|  |  | betacellobiosidase activity |
|  |  | betaglucosidase activity |
|  |  | betaxylosidase activity |
|  |  | microbial peroxidase activity |
|  |  | microbial respiration |
|  |  | phenoloxidase activity |
|  | microbial N content | microbial N content |
|  | microbial N cycle | LAP activity |
|  |  | microbial acquisition |
|  |  | NAG activity |
|  |  | nitrogenase activity |
|  | microbial P content | microbial P content |
|  | microbial P cycle | acid phosphatase activity |
|  |  | phosphatase activity |
|  |  | phosphodiesterase activity |
| plant | lichen depth | lichen depth |
|  | litter abundance | cover litter |
|  |  | litter abundance |
|  | litter depth | litter depth |
|  | moss depth | moss depth |
|  | plant abundance belowground | belowground abundance |
|  |  | belowground biomass |
|  | plant abundance bryophytes | aboveground biomass |
|  |  | abundance |
|  |  | abundance change |
|  |  | cover |
|  |  | cover change |
|  |  | occurrence |
|  |  | relative abundance |
|  | plant abundance cryptogams | aboveground biomass |
|  | plant abundance cryptogams and other | cover |
|  | plant abundance deciduous shrubs | aboveground biomass |
|  |  | abundance |
|  |  | cover |
|  | plant abundance dwarf shrubs | aboveground biomass |
|  |  | aboveground biomass leaves |
|  |  | aboveground biomass woody |
|  |  | abundance |
|  |  | abundance change |
|  |  | biomass change |
|  |  | cover |
|  |  | cover change |
|  |  | occurrence |
|  |  | relative abundance |
|  |  | relative cover |
|  |  | stem density |
|  | plant abundance forbs | aboveground biomass |
|  |  | abundance |
|  |  | abundance change |
|  |  | biomass change |
|  |  | cover |
|  |  | occurrence |
|  |  | relative abundance |
|  |  | seedling density |
|  |  | stem density |
|  | plant abundance forbs and shrubs | aboveground biomass |
|  |  | cover |
|  |  | stem density |
|  | plant abundance fungi | cover |
|  | plant abundance graminoids | aboveground biomass |
|  |  | abundance |
|  |  | abundance change |
|  |  | biomass change |
|  |  | cover |
|  |  | occurrence |
|  |  | relative abundance |
|  |  | relative cover |
|  |  | stem density |
|  | plant abundance lichens | aboveground biomass |
|  |  | abundance |
|  |  | abundance change |
|  |  | cover |
|  |  | cover change |
|  |  | occurrence |
|  |  | relative abundance |
|  | plant abundance mosses and lichens | cover |
|  | plant abundance tall shrubs | aboveground biomass |
|  |  | aboveground biomass leaves |
|  |  | aboveground biomass woody |
|  |  | abundance |
|  |  | cover |
|  |  | occurrence |
|  | plant abundance total | aboveground biomass |
|  |  | abundance |
|  |  | abundance change |
|  |  | biomass change |
|  |  | cover |
|  |  | cover change |
|  |  | seedling density |
|  |  | stem density |
|  |  | sward height |
|  | plant abundance trees | occurrence |
|  | plant abundance vascular cryptogams | aboveground biomass |
|  |  | cover |
|  | plant abundance woody species | aboveground biomass |
|  |  | cover |
|  | plant C content | C pool |
|  |  | C pool below |
|  |  | organic matter content |
|  |  | plant C content |
|  |  | plant C content below |
|  | plant CN ratio | CN ratio |
|  |  | CN ratio below |
|  | plant competition intensity | competition intensity |
|  | plant defense | catechin content |
|  |  | condensed tannins |
|  |  | ellagitannin content |
|  |  | flavonoid content |
|  |  | galloylglucose content |
|  |  | hydrolysable tannin content |
|  |  | phenolic content |
|  |  | quinic acid derivatives content |
|  |  | Si:N ratio |
|  |  | Si:P ratio |
|  |  | Si content |
|  |  | volatile organic compounds |
|  | plant diversity | community stability |
|  |  | diversity |
|  |  | species frequency |
|  |  | species gained |
|  |  | species lost |
|  |  | species richness change |
|  | plant diversity variability | community dissimilarity |
|  |  | richness variability |
|  | plant evenness | dominance |
|  |  | evenness |
|  | plant fitness | flower density |
|  |  | flower new |
|  |  | flower weight |
|  |  | flowering change |
|  |  | flowering frequency |
|  |  | flowering individuals |
|  |  | nr flowering shoots |
|  |  | nr flowers |
|  |  | nr inflorescences |
|  |  | nr seeds |
|  | plant height | height |
|  | plant leaf size | leaf area |
|  |  | leaf area index |
|  |  | leaf biomass |
|  |  | leaf length |
|  |  | leaf weight |
|  |  | leaf width |
|  |  | specific leaf area |
|  | plant microclimate | moss layer moisture |
|  |  | moss layer temperature |
|  | plant minerals | Ca content |
|  |  | Cl content |
|  |  | K content |
|  |  | Mg content |
|  |  | Na content |
|  | plant N content | N content below |
|  |  | N pool |
|  |  | plant N content |
|  | plant NP ratio | NP ratio |
|  | plant P content | P content |
|  | plant physiology | acidic polyphenoloxidase activity |
|  |  | alkaline polyphenoloxidase activity |
|  |  | anthocyanin content |
|  |  | catalase activity |
|  |  | chlorophyll content |
|  |  | chlorophyll fluorescence |
|  |  | evotranspiration |
|  |  | leaf greenness |
|  |  | peroxidase activity |
|  |  | polyphenoloxidase/peroxidase activity |
|  |  | stomatal conductance |
|  |  | water use efficiency |
|  | plant population dynamics | colonization |
|  |  | extinction |
|  |  | germination |
|  |  | mortality |
|  |  | nr seedlings |
|  |  | seed recruitment |
|  |  | survival |
|  | plant population structure | age |
|  |  | sex ratio |
|  | plant quality | acid detergent fibre |
|  |  | cellulose |
|  |  | crude protein |
|  |  | digestibility |
|  |  | energy content |
|  |  | free aminoacids |
|  |  | glucose and fructose content |
|  |  | gross energy |
|  |  | lignin |
|  |  | protein-bound aminoacids content |
|  |  | ratio CP:ADF |
|  |  | soluble sugar content |
|  |  | starch content |
|  |  | sucrose content |
|  |  | sugar content |
|  |  | sugar content below |
|  |  | TNC content |
|  |  | TNC content below |
|  | plant species accumulation rate | species accumulation rate |
|  | plant species richness | seedling richness |
|  |  | species richness |
|  | plant structure | dead biomass |
|  |  | diameter |
|  |  | leaf:shoot ratio |
|  |  | leaf density |
|  |  | live biomass |
|  |  | nr buds |
|  |  | nr internodes |
|  |  | nr leaves |
|  |  | nr shoots |
|  |  | root:shoot ratio |
|  |  | shoot length |
|  |  | shoot weight |
|  |  | total biomass |
|  | plant structure belowground | bulb biomass |
|  |  | nr roots |
|  |  | rhizome length |
|  |  | root biomass |
|  |  | rooting depth |
|  | plant structure change | height change |
|  |  | leaf change |
|  |  | leaf death |
|  |  | leaf growth |
|  |  | leaves new |
|  |  | ring width |
|  |  | root growth |
|  |  | shoot change |
|  |  | shoot death |
|  |  | shoot dieback |
|  |  | shoot growth |
|  |  | shoot new |
|  | vegetation structure | bare ground cover |
| soil | albedo | albedo |
|  | depth organic horizon | depth organic horizon |
|  | microorganism abundance | microorganism abundance |
|  |  | microorganism relative abundance |
|  | microorganism diversity | microorganism diversity |
|  |  | microorganism richness |
|  | N immobilization | N immobilization |
|  |  | N microbial uptake |
|  | N mineralization | N mineralization |
|  | non-polar extractives | non-polar extractives |
|  | permafrost depth | active layer depth |
|  |  | permafrost depth |
|  |  | thaw depth |
|  | pH | pH |
|  | phenolic content soils | soil phenolic content |
|  | redox conditions | cation exchange capacity |
|  |  | CH4 content |
|  |  | O2 concentration |
|  |  | redox potential |
|  |  | soil free aminoacids |
|  | salinity | salinity |
|  | soil C change | soil C change |
|  | soil C labile | acetate content |
|  |  | acid-soluble fraction |
|  |  | organic matter |
|  |  | soil C labile |
|  | soil C total | soil C pool |
|  |  | soil C total |
|  | soil CN ratio | soil CN ratio |
|  | soil compaction | air saturation |
|  |  | bulk density |
|  | soil depth | depth |
|  |  | thickness |
|  | soil moisture | soil moisture |
|  | soil N labile | soil N labile |
|  | soil N total | soil N total |
|  | soil organic acids | acid-insoluble residue |
|  |  | total organic acids |
|  | soil P total | soil P content |
|  | soil respiration | soil respiration |
|  | soil temperature | soil temperature |
|  | water table depth | saturated depth |
|  |  | water table depth |

## Plant functional groups

**Table S4.3.** Plant functional groups used in the systematic review and plant ID reported by the studies, grouped by type (whether they referred to species or groups of species)

| **plant group** | **type** | **reported plant ID** |
| --- | --- | --- |
| bryophytes | species | *Aulacomnium palustre* |
|  |  | *Aulacomnium spp* |
|  |  | *Barbilophozia kunzeana* |
|  |  | *Calliergon richardsonii* |
|  |  | *Conostomum tetragonum* |
|  |  | *Cynodontium spp* |
|  |  | *Hylocomium splendens* |
|  |  | *Kiaeria spp* |
|  |  | *Kiaeria starkei* |
|  |  | *Lophozia spp* |
|  |  | *Marchantiophyta* |
|  |  | *Mnium spp* |
|  |  | *Moerkia blyttii* |
|  |  | *Paludella squarrosa* |
|  |  | *Phlia/Bryum* |
|  |  | *Plagiomnium spp* |
|  |  | *Pleurozium schreberi* |
|  |  | *Pohlia spp* |
|  |  | *Polytrichastrum alpinum* |
|  |  | *Polytrichastrum sexangulare* |
|  |  | *Polytrichum alpinum* |
|  |  | *Polytrichum commune* |
|  |  | *Polytrichum hyperboreum* |
|  |  | *Polytrichum juniperinum* |
|  |  | *Polytrichum juniperium* |
|  |  | *Polytrichum norvegicum* |
|  |  | *Polytrichum piliferum* |
|  |  | *Polytrichum spp* |
|  |  | *Racomitrium lanuginosum* |
|  |  | *Sanionia spp* |
|  |  | *Sanionia uncinata* |
|  |  | *Sphagnum spp* |
|  |  | *Straminergon stramineum* |
|  |  | *Warnstorfia spp* |
|  | group | Bryales |
|  |  | Bryidae |
|  |  | bryophytes |
|  |  | colonizing bryophytes |
|  |  | Dicranum spp and Kiaeria spp |
|  |  | Gymnomitrion type hepatics |
|  |  | hepatics |
|  |  | mosses |
|  |  | Polytrichaceae |
|  |  | robust bryophytes |
|  |  | small bryophytes |
| cryptogams | group | cryptogams |
| cryptogams and other | group | cryptogams and other |
| dwarf shrubs | species | *Andromeda polifolia* |
|  |  | *Betula nana* |
|  |  | *Cassiope hypnoides* |
|  |  | *Dryas octopetala* |
|  |  | *Empetrum hermaphroditum* |
|  |  | *Empetrum nigrum* |
|  |  | *Ledum decumbens* |
|  |  | *Ledum groenlandicum* |
|  |  | *Phyllodoce caerulea* |
|  |  | *Rhododendron subarcticum* |
|  |  | *Salix arctica* |
|  |  | *Salix herbacea* |
|  |  | *Salix polaris* |
|  |  | *Salix polaris female* |
|  |  | *Salix polaris male* |
|  |  | *Salix uva-ursi* |
|  |  | *Vaccinium caespitosum* |
|  |  | *Vaccinium myrtillus* |
|  |  | *Vaccinium uliginosum* |
|  |  | *Vaccinium vitis-idaea* |
|  | group | deciduous dwarf shrubs |
|  |  | dwarf shrubs |
|  |  | ericoids |
|  |  | ericoids and evergreen shrubs |
|  |  | evergreen dwarf shrubs |
|  |  | evergreen shrubs |
|  |  | short shrubs |
| forbs | species | *Achillea millefolia* |
|  |  | *Alchemilla spp* |
|  |  | *Bistorta vivipara* |
|  |  | *Campanula rotundifolia* |
|  |  | *Cardamine pratensis* |
|  |  | *Cardamine pratensis subsp. polemonioides* |
|  |  | *Cerastium arcticum* |
|  |  | *Cerastium fontanum* |
|  |  | *Cochlearia groenlandica* |
|  |  | *Equisetum arvense* |
|  |  | *Equisetum pratense* |
|  |  | *Equisetum spp* |
|  |  | *Geranium sylvaticum* |
|  |  | *Gnaphalium norvegicum* |
|  |  | *Gnaphalium supinum* |
|  |  | *Hippurus tetraphylla* |
|  |  | *Linnaea borealis* |
|  |  | *Oxyria digyna* |
|  |  | *Oxytropis viscida* |
|  |  | *Parnassia palustris* |
|  |  | *Plantago maritima* |
|  |  | *Polygonum viviparum* |
|  |  | *Potentilla crantzii* |
|  |  | *Potentilla egedii* |
|  |  | *Ranunculus acris* |
|  |  | *Ranunculus cymbalaria* |
|  |  | *Ranunculus hyperboreus* |
|  |  | *Ranunculus spp* |
|  |  | *Rubus arcticus* |
|  |  | *Rubus chamaemorus* |
|  |  | *Rumex acetosa* |
|  |  | *Rumex acetosella* |
|  |  | *Saussurea alpina* |
|  |  | *Saxifraga cernua* |
|  |  | *Saxifraga cespitosa* |
|  |  | *Saxifraga oppositifolia* |
|  |  | *Sibbaldia procumbens* |
|  |  | *Silene acaulis* |
|  |  | *Solidago macrophylla* |
|  |  | *Solidago virgaurea* |
|  |  | *Stellaria graminea* |
|  |  | *Stellaria longipes* |
|  |  | *Trientalis europaea* |
|  |  | *Trollius europaeus* |
|  |  | *Veronica alpina* |
|  |  | *Veronica longifolia* |
|  |  | *Viola biflora* |
|  |  | *Viola epipsila* |
|  | group | Caryophyllaceae |
|  |  | dicotyledoneous plants |
|  |  | erect herbs |
|  |  | flowering plants |
|  |  | forbs |
|  |  | herbaceous plants |
|  |  | horsetails |
|  |  | Polygonaceae |
|  |  | prostrate herbs |
| forbs and shrubs | group | forbs and shrubs |
| fungi | group | fungi |
| graminoids | species | *Anthoxanthum odoratum* |
|  |  | *Anthoxanthum odoratum spp. alpinum* |
|  |  | *Arctagrostis latifolia* |
|  |  | *Calamagrostis canadensis* |
|  |  | *Calamagrostis deschampsioides* |
|  |  | *Calamagrostis lapponica* |
|  |  | *Calamagrostis purpurea* |
|  |  | *Carex aquatilis* |
|  |  | *Carex aquatilis subsp. stans* |
|  |  | *Carex bigelowii* |
|  |  | *Carex brunnescens* |
|  |  | *Carex glareosa* |
|  |  | *Carex juncella* |
|  |  | *Carex lachenalii* |
|  |  | *Carex membranacea* |
|  |  | *Carex ramenskii* |
|  |  | *Carex spp* |
|  |  | *Carex stans* |
|  |  | *Carex subspathacea* |
|  |  | *Carex vaginata* |
|  |  | *Dactylina arctica* |
|  |  | *Deschampsia alpina* |
|  |  | *Deschampsia caespitosa* |
|  |  | *Deschampsia flexuosa* |
|  |  | *Dupontia fisheri* |
|  |  | *Dupontia psilosantha* |
|  |  | *Eleocharis acicularis* |
|  |  | *Eriophorum angustifolium* |
|  |  | *Eriophorum angustifolium ssp triste* |
|  |  | *Eriophorum scheuchzeri* |
|  |  | *Eriophorum spp* |
|  |  | *Eriophorum vaginatum* |
|  |  | *Festuca ovina* |
|  |  | *Festuca rubra* |
|  |  | *Hierochloe alpina* |
|  |  | *Juncus biglumis* |
|  |  | *Juncus filiformis* |
|  |  | *Juncus trifidus* |
|  |  | *Leymus mollis* |
|  |  | *Luzula arcuata* |
|  |  | *Luzula multiflora* |
|  |  | *Luzula multiflora spp. frigida* |
|  |  | *Luzula spicata* |
|  |  | *Poa abbreviata* |
|  |  | *Poa alpina* |
|  |  | *Poa arctica* |
|  |  | *Poa pratensis subsp. alpigena* |
|  |  | *Poa spp* |
|  |  | *Puccinellia phryganodes* |
|  |  | *Triglochin palustris* |
|  | group | Festuca ovina and Deschampsia flexuosa |
|  |  | graminoids |
|  |  | grasses |
|  |  | Juncaceae |
|  |  | Poaceae |
|  |  | rushes |
|  |  | sedges |
| lichens | species | *Alectoria nigricans* |
|  |  | *Alectoria ochroleuca* |
|  |  | *Cetraria cucullata* |
|  |  | *Cetraria delisei* |
|  |  | *Cetraria islandica* |
|  |  | *Cetraria kamczatka* |
|  |  | *Cetraria nivalis* |
|  |  | *Cetraria spp* |
|  |  | *Cetrariella delisei* |
|  |  | *Cladina arbuscula* |
|  |  | *Cladina mitis* |
|  |  | *Cladina rangiferina* |
|  |  | *Cladina spp* |
|  |  | *Cladina stellaris* |
|  |  | *Cladina uncialis* |
|  |  | *Cladonia amaurocrea* |
|  |  | *Cladonia crispata* |
|  |  | *Cladonia digitata* |
|  |  | *Cladonia gracilis* |
|  |  | *Cladonia nigripes* |
|  |  | *Cladonia rangiferina* |
|  |  | *Cladonia spp* |
|  |  | *Cladonia uncialis* |
|  |  | *Lecidea spp* |
|  |  | *Nephroma arcticum* |
|  |  | *Ochrolechia spp* |
|  |  | *Peltigera rufocanus* |
|  |  | *Psoroma hypnorum* |
|  |  | *Sphaerophorus globosus* |
|  |  | *Stereocaulon paschale* |
|  |  | *Stereocaulon spp* |
|  |  | *Thamnolia spp* |
|  | group | crustose lichens |
|  |  | foliose lichens |
|  |  | fruticose lichens |
|  |  | lichens |
| litter | group | dead vascular plants |
|  |  | litter |
| mosses and lichens | group | mosses lichens |
| tall shrubs | species | *Betula glandulosa* |
|  |  | *Cornus canadensis* |
|  |  | *Cornus suecica* |
|  |  | *Salix glauca* |
|  |  | *Salix hastigiata* |
|  |  | *Salix lapponum* |
|  |  | *Salix ovalifolia* |
|  |  | *Salix phylicifolia* |
|  |  | *Salix planifolia* |
|  |  | *Salix spp* |
|  | group | Betula and Salix |
|  |  | deciduous tall shrubs |
|  |  | tall shrubs |
| total | species | community |
|  | group | vascular plants |
| trees | species | *Betula pubescens* |
| vascular cryptogams | species | *Lycopodium spp* |
|  | group | vascular cryptogams |
| woody species | group | deciduous woody plants |
|  |  | deciduous shrubs |
|  |  | shrubs |
|  |  | woody plants |

## Functional groups of herbivores

**Table S4.4.** Functional groups of herbivores used in the systematic review and herbivore ID reported by the studies, grouped by type (whether they referred to species or groups of species). Functional groups of herbivores were defined by Speed et al. (2019) and represent: F1 limnic-habitat associated herbivores, migrating outside the Arctic for winter, undifferentiated guts for which graminoids are an important diet component (waterfowl; paragon *Anser anser*); F2 immobile, burrowing herbivores with hindgut fermenting digestive physiology (paragon *Synaptomys borealis*); and F3 large-bodied facultative-generalist herbivores for which shrubs and lichens are an important diet component (paragon *Lepus timidus*).

| **functional group** | **type** | **reported herbivore ID** |
| --- | --- | --- |
| F1 | species / subspecies | *Anser albifrons* |
|  |  | *Anser albifrons frontalis* |
|  |  | *Anser brachyrhynchus* |
|  |  | *Chen caerulescens* |
|  |  | *Chen caerulescens atlanticus* |
|  |  | *Chen caerulescens caerulescens* |
|  |  | *Branta bernicla* |
|  |  | *Branta bernicla bernicla* |
|  |  | *Branta bernicla nigricans* |
|  |  | *Branta canadensis* |
|  |  | *Branta canadensis interior* |
|  |  | *Branta canadensis minima* |
|  |  | *Branta leucopsis* |
|  | group | geese |
| F2 | species / subspecies | *Dicrostonyx groenlandicus* |
|  |  | *Dicrostonyx rubicatus* |
|  |  | *Dicrostonyx spp* |
|  |  | *Dicrostonyx torquatus* |
|  |  | *Dicrostonyx torquatus groenlandicus* |
|  |  | *Lemmus lemmus* |
|  |  | *Lemmus sibiricus* |
|  |  | *Lemmus spp* |
|  |  | *Lemmus trimucronatus* |
|  |  | *Microtus agrestis* |
|  |  | *Microtus gregalis* |
|  |  | *Microtus middendorffi* |
|  |  | *Microtus miurus* |
|  |  | *Microtus oeconomus* |
|  |  | *Microtus spp* |
|  |  | *Myodes rufocanus* |
|  |  | *Myodes spp* |
|  | group | lemmings |
|  |  | microtine rodents |
|  |  | rodents |
|  |  | small mammals |
|  |  | voles |
| F3 | species / subspecies | *Alces alces* |
|  |  | *Lagopus lagopus* |
|  |  | *Lagopus muta* |
|  |  | *Lagopus muta hyperborea* |
|  |  | *Lagopus spp* |
|  |  | *Lepus americanus* |
|  |  | *Lepus arcticus* |
|  |  | *Lepus timidus* |
|  |  | *Myodes rutilus* |
|  |  | *Ovibos moschatus* |
|  |  | *Ovis aries* |
|  |  | *Rangifer tarandus* |
|  |  | *Rangifer tarandus groenlandicus* |
|  |  | *Rangifer tarandus platyrhynchus* |
|  |  | *Rangifer tarandus tarandus* |
|  |  | *Urocitellus parryii* |
|  | group | large herbivores |
| inv | species / subspecies | *Aculus tetranothix* |
|  |  | *Epirrita autumnata* |
|  |  | *Euura aquilonis* |
|  |  | *Euura arctica* |
|  |  | *Operophtera brumata* |
|  |  | *Pontania nivalis* |
|  | group | defoliating invertebrates |
|  |  | eriophyid mites |
|  |  | galling invertebrates |
|  |  | invertebrate herbivores |
|  |  | mining invertebrates |

# Additional analyses using alternative approaches

In the analyses presented in the main text we made assumptions regarding the grouping of herbivores into functional groups and the occurrence of extreme values. In this section we present the implications of each approach and compare the analyses using alternative approaches to the ones presented in the main text.

## Analyses grouping herbivores depending on their body size

The main analyses in the manuscript grouped herbivores based on functional groups identified by (Speed et al., 2019). A simpler approach is to group herbivores based on their body size, an important trait that determines their impacts on ecosystems (Bakker et al., 2006; Legagneux et al., 2014). Here we report the results of analyses based on grouping herbivores depending on their body size (**Table S4.5**).

**Table S4.5.** Body size group of herbivores used in the systematic review and herbivore ID reported by the studies, grouped by type (whether they referred to species or groups of species).

| **body size group** | **type** | **reported herbivore ID** |
| --- | --- | --- |
| invertebrate herbivores | species / subspecies | *Aculus tetranothix* |
|  |  | *Epirrita autumnata* |
|  |  | *Euura aquilonis* |
|  |  | *Euura arctica* |
|  |  | *Operophtera brumata* |
|  |  | *Pontania nivalis* |
|  | group | defoliating invertebrates |
|  |  | eriophyid mites |
|  |  | galling invertebrates |
|  |  | invertebrate herbivores |
|  |  | mining invertebrates |
| large herbivores | species / subspecies | *Alces alces* |
|  |  | *Ovibos moschatus* |
|  |  | *Ovis aries* |
|  |  | *Rangifer tarandus* |
|  |  | *Rangifer tarandus groenlandicus* |
|  |  | *Rangifer tarandus platyrhynchus* |
|  |  | *Rangifer tarandus tarandus* |
|  | group | large herbivores |
| medium herbivores | species / subspecies | *Anser albifrons* |
|  |  | *Anser albifrons frontalis* |
|  |  | *Anser brachyrhynchus* |
|  |  | *Chen caerulescens* |
|  |  | *Chen caerulescens atlanticus* |
|  |  | *Chen caerulescens caerulescens* |
|  |  | *Branta bernicla* |
|  |  | *Branta bernicla bernicla* |
|  |  | *Branta bernicla nigricans* |
|  |  | *Branta canadensis* |
|  |  | *Branta canadensis interior* |
|  |  | *Branta canadensis minima* |
|  |  | *Branta leucopsis* |
|  |  | *Lagopus lagopus* |
|  |  | *Lagopus muta* |
|  |  | *Lagopus muta hyperborea* |
|  |  | *Lagopus spp* |
|  |  | *Lepus americanus* |
|  |  | *Lepus arcticus* |
|  |  | *Lepus timidus* |
|  |  | *Urocitellus parryii* |
|  | group | geese |
| small herbivores | species / subspecies | *Dicrostonyx groenlandicus* |
|  |  | *Dicrostonyx rubicatus* |
|  |  | *Dicrostonyx spp* |
|  |  | *Dicrostonyx torquatus* |
|  |  | *Dicrostonyx torquatus groenlandicus* |
|  |  | *Lemmus lemmus* |
|  |  | *Lemmus sibiricus* |
|  |  | *Lemmus spp* |
|  |  | *Lemmus trimucronatus* |
|  |  | *Microtus agrestis* |
|  |  | *Microtus gregalis* |
|  |  | *Microtus middendorffi* |
|  |  | *Microtus miurus* |
|  |  | *Microtus oeconomus* |
|  |  | *Microtus spp* |
|  |  | *Myodes rufocanus* |
|  |  | *Myodes rutilus* |
|  |  | *Myodes spp* |
|  | group | lemmings |
|  |  | microtine rodents |
|  |  | rodents |
|  |  | small mammals |
|  |  | voles |

**Contrasts based on herbivore body size groups.** The most common contrasts in herbivore diversity were exclusion studies of medium sized mammals (medium sized herbivores vs zero; 59 articles, 733 studies) and large herbivores (large herbivores vs zero; 53 articles, 834 studies, **Table S4.6**), followed by exclusion of large and small herbivores (24 articles, 313 studies).

**Table S4.6** Herbivore diversity contrasts considered in the systematic review, based on body size groups of herbivores. Numerical change indicates the difference in groups between high and low diversity areas reported in each study. Identity of change describes which group of herbivores differed between high and low diversity areas. Herbivore diversity contrast specifies the groups of herbivores present in high and low diversity areas (high | low). Number of records indicates how many articles and studies (in brackets) reported each type of contrast.

| **numerical change** | **identity of change** | **herbivore diversity contrast** | **nr of records** |
| --- | --- | --- | --- |
| 0 | no contrast (zero) | invertebrate herbivores \| invertebrate herbivores | 3(16) |
|  |  | large herbivores \| medium herbivores | 1(6) |
|  |  | small herbivores \| large herbivores | 1(4) |
| 1 | invertebrate herbivores (Inv) | invertebrate herbivores \| zero | 14(150) |
|  | large herbivores (L) | invertebrate herbivores; large herbivores; medium herbivores \| invertebrate herbivores; medium herbivores | 1(2) |
|  |  | invertebrate herbivores; large herbivores; medium herbivores; small herbivores \| invertebrate herbivores; medium herbivores; small herbivores | 2(19) |
|  |  | large herbivores \| zero | 53(834) |
|  |  | large herbivores; medium herbivores \| medium herbivores | 1(3) |
|  |  | large herbivores; medium herbivores; small herbivores \| medium herbivores; small herbivores | 7(83) |
|  | medium herbivores (M) | invertebrate herbivores; medium herbivores \| invertebrate herbivores | 1(2) |
|  |  | large herbivores; small herbivores \| small herbivores | 14(406) |
|  |  | medium herbivores \| zero | 59(733) |
|  |  | medium herbivores; small herbivores \| small herbivores | 3(20) |
|  | small herbivores (S) | small herbivores \| zero | 20(379) |
| 2 | large and medium herbivores (LM) | invertebrate herbivores; large herbivores; medium herbivores \| invertebrate herbivores | 1(2) |
|  |  | large herbivores; medium herbivores \| zero | 9(107) |
|  |  | large herbivores; medium herbivores; small herbivores \| small herbivores | 7(274) |
|  | large and small herbivores (LS) | large herbivores; small herbivores \| zero | 24(313) |
|  | medium and small (MS) | medium herbivores; small herbivores \| zero | 6(47) |
| 3 | large medium and small herbivores (LMS) | invertebrate herbivores; large herbivores; medium herbivores; small herbivores \| invertebrate herbivores | 1(27) |
|  |  | large herbivores; medium herbivores; small herbivores \| zero | 17(286) |

## Analyses without removing extreme values

In the main analyses presented in the manuscript data were truncated to include 99% of the observations because of extreme values generated mainly from the imputation of missing SD values. In the complete dataset, effect sizes ranged between -6893.7 to 11841.9. The truncated dataset excluded 36 studies (34 of them resulting from imputed SD values), and the effect sizes ranged between -22.0 and 8.2. To make sure that removing these extreme values did not affect our results we ran the analyses including those values (EV in **Figure S4.1** and **S4.2**).

## Comparison of analyses using different approaches

The number of studies included in each approach differed slightly (**Figure S4.1**).

For outcome variables reported by more than 5 articles, we ran intercept-only models. For the analyses not excluding extreme values (EV) this step involved the same 47 outcome variables as included in the main text. For the analyses grouping herbivores depending on their body size (BS), this included 48 variables (plant structure belowground was not included in the other analyses). The results for the intercept-only models were largely consistent for the three approaches, except in the case of plant C content which was significantly affected by herbivore diversity when extreme values were not removed (**Figure S4.2**). For outcome variables reported by more than 10 articles, we ran multi-moderator meta-regression models for the three approaches, which broadly yielded similar results (**Table S4.7**).

**Figure S4.1** Number of studies included in the systematic review database, the overall quantitative synthesis and the meta-regressions in the main text (main), the analyses not removing extreme values (EV) and the analyses grouping herbivores based on their body size (BS).


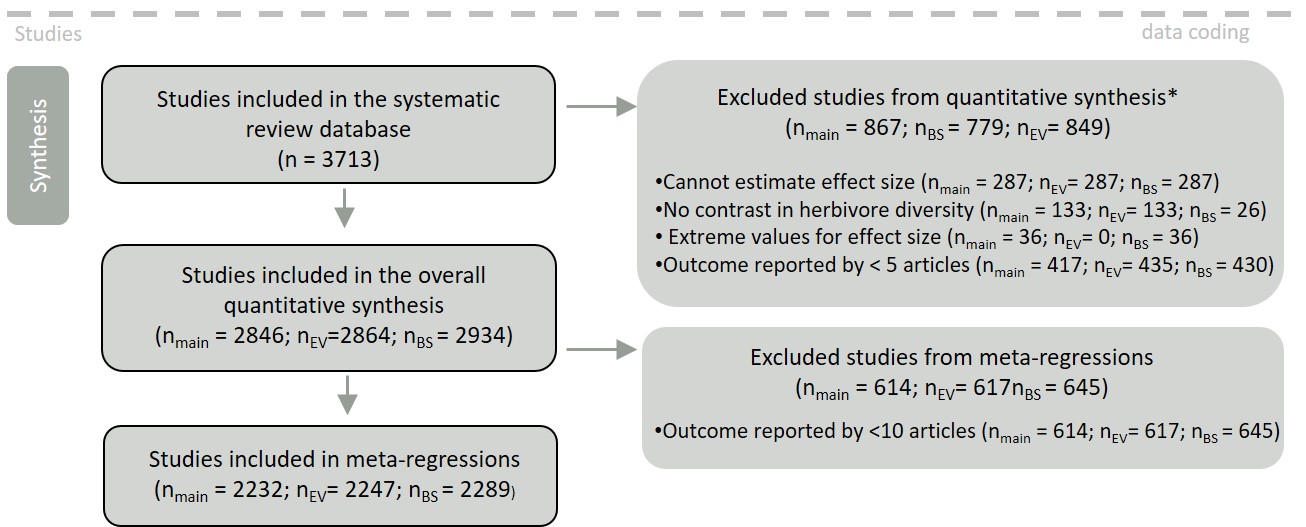


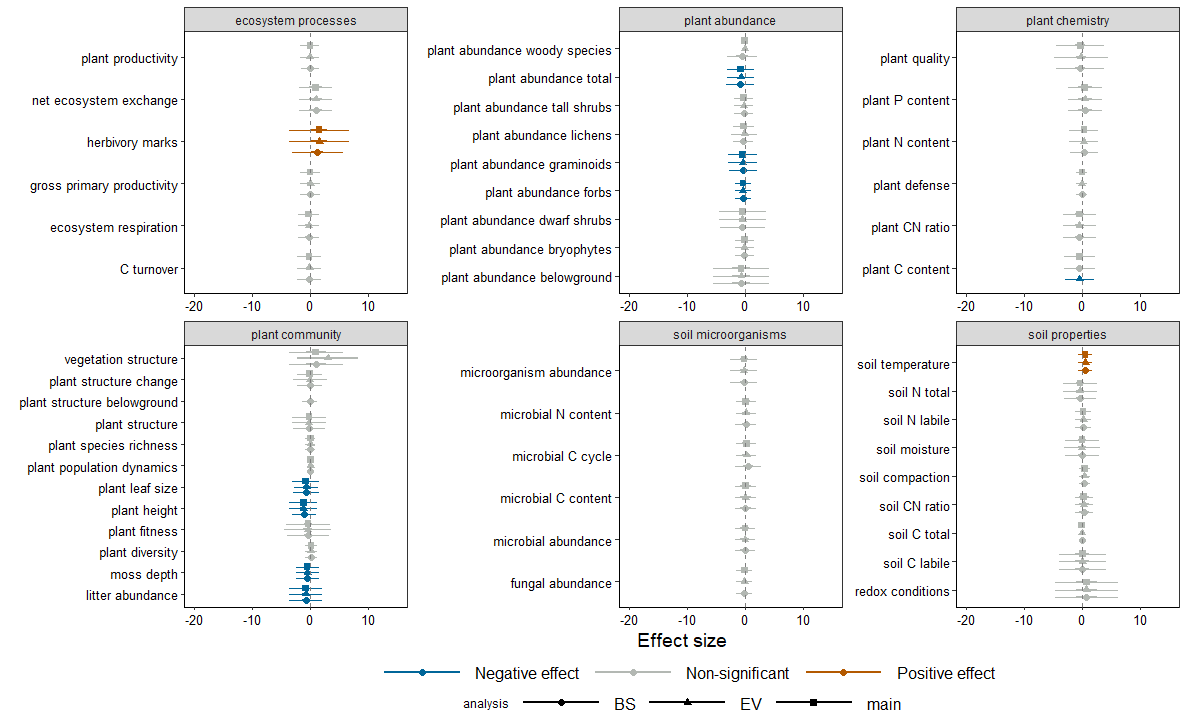
**Figure S4.2** Effect of the contrast in herbivore diversity on outcome variables reported by at least 5 articles, for analyses included in the main text (main), the analyses not removing extreme values (EV) and the analyses grouping herbivores based on their body size (BS). Symbols represent overall effect sizes with colour indicating the significance and direction of the effect. Thin lines represent prediction intervals and thicker lines (often hidden behind the overall effect size) represent confidence intervals.

**Table S4.7** Comparison of multi-moderator meta-regression models for the three approaches: for analyses included in the main text (main), the analyses not removing extreme values (EV) and the analyses grouping herbivores based on their body size (BS). Outcome variables in bold indicate those that had a significant effect. Rows highlighted in grey indicate that the models were the same for the different approaches.

| **Outcome variable** | **Main** | **EV** | **BS** |
| --- | --- | --- | --- |
| plant CN ratio | yi_smd ~ **change_f** + **exclusion** + spatial_resolution | yi_smd ~ **change_f** + **exclusion** + spatial_resolution | yi_smd ~ **exclusion** + spatial_resolution |
| plant C content | yi_smd ~ **overall_criterion** | -- | yi_smd ~ **overall_criterion** |
| abundance bryophytes | yi_smd ~ exclusion | yi_smd ~ **exclusion** | -- |
| abundance dwarf shrubs | yi_smd ~ herb_fgr_change + exclusion + spatial resolution | yi_smd ~ herb_fgr_change + exclusion + spatial_resolution | yi_smd ~ change + exclusion + **spatial_resolution** |
| abundance forbs | -- | yi_smd ~ overall_criterion + **spatial_resolution** | -- |
| abundance graminoids | yi_smd ~ **change_f** | yi_smd ~ **change_f** | yi_smd ~ **change** |
| abundance lichens | yi_smd ~ **change_f** | yi_smd ~ **exclusion** + **study_length** | yi_smd ~ **change** |
| abundance tall shrubs | yi_smd ~ **error_type** | yi_smd ~ **error_type** | -- |
| plant diversity | yi_smd ~ distance_to_treeline + **habitat_type** | yi_smd ~ distance_to_treeline + **habitat_type** | yi_smd ~ distance_to_treeline + **habitat_type** |
| plant fitness | yi_smd ~ **error_type** | yi_smd ~ **error_type** | yi_smd ~ **error_type** |
| plant height | yi_smd ~ **change_f** + **error_type** + exclusion + habitat_type + overall_criterion + **study_length** | yi_smd ~ change_f + **error_type** + exclusion + habitat_type + overall_criterion + **study_length** | yi_smd ~ **change** + **error_type** + **exclusion** + habitat_type + overall_criterion + study_length |
| plant leaf size | -- | yi_smd ~ **habitat_type** | -- |
| plant productivity | yi_smd ~ permafrost_ord+ recent_warming + temperature | yi_smd ~ permafrost_ord + recent_warming + temperature | yi_smd ~ permafrost_ord + temperature + recent_warming |
| plant species richness | -- | -- | yi_smd ~ habitat_type |
| plant structure | yi_smd ~ **year.c** | yi_smd ~ **year.c** | yi_smd ~ **year.c** |
| soil C labile | yi_smd ~ error_type | yi_smd ~ error_type | yi_smd ~ error_type |
| soil moisture | yi_smd ~ **error_type** + **year.c** | yi_smd ~ **error_type** + **year.c** | yi_smd ~ **error_type** + **year.c** |

# Ecological modifiers of the effect of herbivore diversity

The effects of herbivore diversity can be modulated by local environmental conditions. These key sources of heterogeneity were identified based on expert knowledge and discussions with relevant stakeholders in the protocol development team (Barrio et al., 2022; **Table S4.8**). These variables were extracted from the studies during the coding process or from existing data layers (see references in **Table S4.8**) to ensure that the information on these variables was extracted in a consistent way across studies. Collinearity among variables was assessed using Pearson correlation with r>|0.7| as a threshold for excluding a variable.

**Table S4.8.** List of potential ecological modifiers for the effects of herbivore diversity on tundra ecosystems. Type indicates whether the variable is numerical (num) or categorical (cat). Source indicates where the data is extracted from: P for publication, D for digital spatial data layers and C classified by the reviewers based on information available in the publication. Number of missing data (NAs) or not reported values in the database (number of studies out of 2235 included in meta-regressions)

| **ecological modifier** | **description** | **type** | **source** | **NAs in database** |
| --- | --- | --- | --- | --- |
| elevation_DEM | elevation above mean sea level (m) extracted from digital elevation model used for creation of the Circumpolar Arctic Vegetation Map [1]; areas outside this map extracted from ASTER Global Digital Elevation Map [2] | num | D | 79 |
| distance_to_treeline | geodesic distance (km) to meridional limit of arctic subzone E, based on [1] | num | D | 79 |
| distance_from_coast | geodesic distance (km) to coast, based on the coastline and islands map used in the creation of the Circumpolar Arctic Vegetation Map [1] | num | D | 79 |
| bioclimatic_zone | Circumpolar Arctic Region Bioclimate Subzones according to [1] | num | D | 81 |
| temperature | mean summer temperature (June-August, °C), based on WorldClim 30-sec. data [3] | num | D | 128 |
| precipitation | mean annual precipitation (mm), based on WorldClim 30-sec. data [3] | num | D | 128 |
| growing_season | duration of growing season (days), according to [4] | num | D | 491 |
| productivity | value of NDVI (vegetation greenness), according to [4] | num | D | 491 |
| recent_warming | extent of change in mean temperature (°C) from 1951–1980 to 2000–2020 according to [5, 6] | num | D | 79 |
| recent_greening | extent of change in cumulative daily growing season NDVI (% per decade) in 1982–2014 according to [4] | num | D | 449 |
| extent_of_recent_change | extent of recent change in growing season length according to [4] | num | D | 449 |
| soil_chemistry | soil chemistry | cat | C | 2062 |
| soil_texture | broad categories of soil texture | cat | C | 2137 |
| soil_moisture | soil moisture as described in the study | cat | C | 1340 |
| soil_type | soil type as reported by the authors | cat | P | 1319 |
| soil_type_D | soil type according to the Circumpolar Arctic Substrate Chemistry map used in the creation of Circumpolar Arctic Vegetation Map [1] | cat | D | 182 |
| permafrost | presence and type of permafrost | cat | C | 1759 |
| permafrost_D | extent of permafrost according to [7], with categories grouped into no permafrost, isolated patches, sporadic, discontinuous and continuous permafrost; treated as an ordinal variable (permafrost_ord) with values between 0 and 4 | num | D | 80 |
| habitat_type | habitat types using the broad categories defined in CAVM | cat | C | 47 |
| habitat_type_D | habitat types, according to [1] | cat | D | 1355 |

[1] CAVM Team. (2003). Circumpolar Arctic Vegetation Map. Scale 1:7,500,000. Conservation of Arctic Flora and Fauna (CAFF) Map No. 1. U.S. Fish and Wildlife Service, Anchorage, Alaska.

[2] Advanced Spaceborne Thermal Emission and Reflection Radiometer (ASTER) Global Digital Elevation Model Version 3 (GDEM 003), <https://asterweb.jpl.nasa.gov/gdem.asp>

[3] Fick, S. E., Hijmans, R. J. (2017). WorldClim 2: new 1‐km spatial resolution climate surfaces for global land areas. International Journal of Climatology, 37(12), 4302-4315. Speed et al. in prep (for the PCA axes of variation).

[4] Park, T., Ganguly, S., Tømmervik, H., Euskirchen, E. S., Høgda, K. A., Karlsen, S. R., ... Myneni, R. B. (2016). Changes in growing season duration and productivity of northern vegetation inferred from long-term remote sensing data. Environmental Research Letters, 11(8), 084001.

[5] GISTEMP Team, 2016: GISS Surface Temperature Analysis (GISTEMP). NASA Goddard Institute for Space Studies.

[6] Hansen, J., Ruedy, R., Sato, M., Lo, K. (2010). Global surface temperature change, Rev. Geophys., 48, RG4004, doi:10.1029/2010RG000345.

[7] Brown, J., Ferrians, O., Heginbottom, J.A., Melnikov, E. (2002). Circum-Arctic Map of Permafrost and Ground-Ice Conditions, Version 2. Boulder, Colorado USA. NSIDC: National Snow and Ice Data Center. <https://doi.org/10.7265/skbg-kf16>

Some of these ecological modifiers were strongly correlated (**Figure S4.3**) and/or had a large number of missing values (**Table S4.8**). The strongest correlation was found between recent_greening and extent_of_recent_change (r = 0.93), so we kept recent_greening because it had weaker correlations to other variables. Productivity, temperature, growing season and bioclimatic zone were also correlated (r>0.7); we kept temperature because it had the lower number of missing values. Thus, the ecological modifiers considered in our final analyses were: elevation, geodesic distance to treeline and to the coast, mean annual temperature, mean annual precipitation, recent warming, recent greening, soil type, extent of permafrost and habitat type.

**Figure S4.3** Correlation matrix between numerical ecological modifiers. Pearson’s correlation (r) values are shown, with colours indicating strength and direction of the correlation.


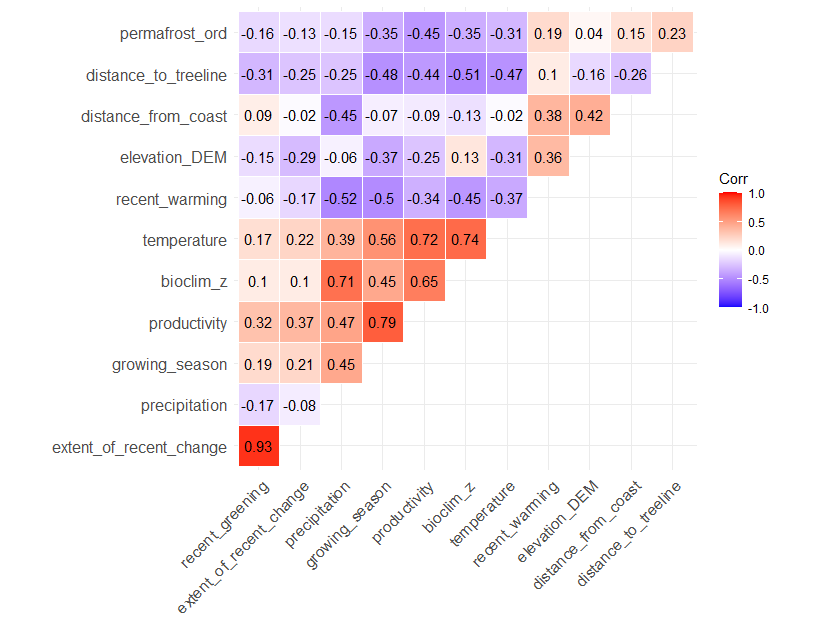


# Herbivore diversity vs herbivore exclusion?

For many studies the contrast in herbivore diversity was the comparison of areas with some herbivores to areas with no herbivores (i.e., complete exclusion, in contrast to partial exclusion studies where some groups of herbivores were present in the low diversity areas). Thus, the interpretation of the effect of herbivore diversity could be confounded with the effect of herbivore exclusion. To check if this was the case, we re-ran the intercept-only models removing studies for which the low level of herbivore diversity was zero (2,337 studies; 82.1%). After removing these studies (i.e., including only partial exclusion studies), only 5 variables were measured by at least 5 articles: plant height, total plant abundance and the abundance of lichens, graminoids and dwarf shrubs. Three of these outcome variables (plant height, total plant abundance and abundance of graminoids) responded significantly to the contrast in herbivore diversity when the whole dataset was included, but these effects did not remain significant when studies for which the low level of herbivore diversity was zero (i.e., studies excluding all herbivores). Two of the outcome variables (abundance of lichens and dwarf shrubs) did not respond significantly to the contrast in herbivore diversity neither when the whole dataset was included nor when only partial exclusion studies were considered. These results suggest that those effects are more likely driven by herbivore exclusion than by herbivore diversity itself, and our results should be interpreted with caution.

**Figure S4.4** Results of intercept-only models when studies for which the low level of herbivore diversity was zero (i.e., complete exclusion studies) were removed from the dataset. No significant effects were found for any of the outcome variables reported by more than 5 articles. Variables that changed between the analysis including the whole dataset and the analysis including only partial exclusion studies are indicated with asterisks beside the variable name.


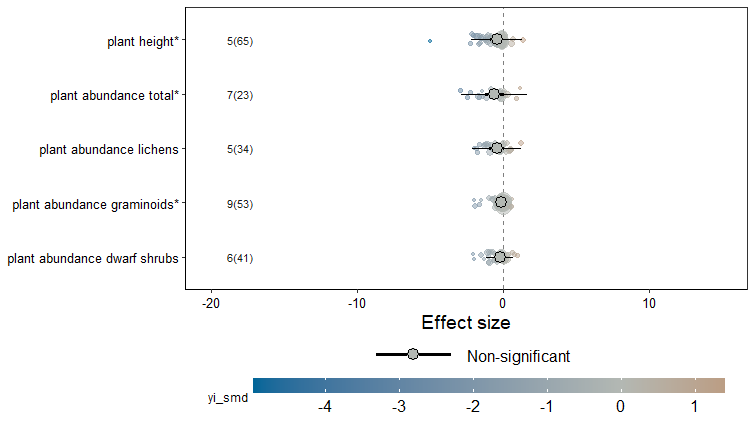


# Studies including size-selective exclosures

Using size-selective exclosures (or factorial exclosures) represents an important tool to study the effects of herbivore diversity (Bakker, 2017). Among the studies included in the systematic review, 636 studies belonging to 15 articles specifically addressed the effect of different groups of herbivores using size-selective exclosures, where different groups of herbivores are excluded sequentially depending on their body size (**Table S4.9**). Of these, 13 articles (480 studies) were included in quantitative syntheses.

**Table S4.9** List of articles included in the systematic review that used size-selective exclosures to address the effects of different groups of herbivores. Types of exclosures: LHe (exclude large herbivores), MHe (exclude large and medium sized herbivores), SHe (exclude large, medium and small herbivores); controls are plots open to all herbivores. The number of studies extracted from each article in the systematic review are indicated, as well as whether the studies were included in quantitative synthesis, meta-regressions or excluded. Articles are arranged by study site, from W to E.

| **Site** | **Exclosures** | **Details** | **Target herbivores** | **Ref** | **Main findings** | **Nr of studies** |
| --- | --- | --- | --- | --- | --- | --- |
| Toolik Lake (Alaska) | LHe, MHe, SHe, control | 2 blocks established in 1989; dry heath tundra | LHe: caribou (Rangifer tarandus); MHe: ground squirrel (*Spermophilus parryii*); SHe: voles (*Microtus spp*), and lemmings (*Lemmus sibiricus* and *Dicrostonyx rubricatus*) | [1] | **Herbivore exclusion** (irrespective of which herbivore group was excluded) reduced litter cover (control > LHe = MHe = SHe) and increased the total aboveground biomass (LHe = MHe = SHe > control). **Additive effect** of herbivore exclusion on biomass of Cladonia (SHe > MHe = LHe > control). | 33 studies (26 included in meta-regressions, 7 excluded because of lack of diversity contrast) |
|  | LHe, SHe, control | 3 blocks established in 1996; dry heath tundra | LHe: caribou (*Rangifer tarandus*); SHe: collared lemming (*Dicrostonyx groenlandicus*), singing vole (*Microtus miurus*) | [2] | **Additive effect** of herbivore exclusion on leaf area index and vegetation abundance (SHe > LHe > control); **compensatory effect** on the abundance of foliose and fruticose lichens and total lichen abundance (LHe > SHe = control). **Herbivore exclusion** (irrespective of which herbivore group was excluded) reduced the amount of bare ground and crustose lichens (control > LHe = SHe) and increased the abundance of vascular plants (LHe = SHe > control). | 30 studies (27 included in meta-regressions, 3 included in quantitative synthesis) |
|  |  |  |  | [3] | **Compensatory effect** of herbivore exclusion on phosphodiesterase activity in soils (LHe > SHe = control), increases in phosphatases suggests microbial P limitation when only large herbivores are excluded. Trends towards compensatory effects were also found for lichens (similar to Min et al. 2021). **No effects** of herbivore exclusion in any other measured variable. | 75 studies (10 included in meta-regressions, 24 included in quantitative synthesis, 25 excluded because of lack of diversity contrast and 16 excluded because they referred to outcome variables reported by less than 5 articles) |
|  |  | 2 blocks established in 1989 in both dry heath tundra (same as [1]) and moist acidic tundra and 3-4 blocks established in 1996 in dry heath tundra (same as [2,3]) and moist acidic tundra | LHe: caribou (*Rangifer tarandus*); SHe: tundra vole (Microtus oeconomus), singing vole (*Microtus miurus*), brown lemmings (Lemmus trimucronatus) and Arctic ground squirrel (Spermophilus parryi) | [4] | **Additive effect** of herbivore exclusion on vascular plant density and the abundance of graminoids (SHe > LHe = control) in moist acidic tundra but not in dry heath. No effect on NDVI or LAI, the abundance of other plant functional groups or plant diversity. **Herbivore exclusion** (irrespective of which herbivore group was excluded) increased the height of deciduous dwarf shrubs in moist acidic tundra and on lichen thickness in dry tundra (control < LHe = SHe). **No effects** of herbivore exclusion in any other measured variable. | 84 studies (72 included in meta-regressions, 6 included in quantitative synthesis, 6 excluded because they referred to outcome variables reported by less than 5 articles) |
| Vássijávri (Sweden) | LHe, SHe, control | 3 replicate blocks established in 1998 | LHe: reindeer (*Rangifer tarandus*); SHe: gray-sided vole (*Myodes rufocanus*), Norwegian lemming (*Lemmus lemmus*) | [5] | (results pooled across sites) **Additive effect** of herbivore exclusion (or effect of small herbivores?) on the relative change in abundance of *Vaccinium myrtillus*, *V. vitis-idaea, Dicranum spp.* and *Polytrichum spp* (control = LHe < SHe) and for *Ptilidium ciliare* (control = LHe > SHe). **Herbivore exclusion** (irrespective of which herbivore group was excluded) increased the relative change in abundance of *Cladina mitis* (control < LHe = SHe). | 3 studies (all included in meta-regressions) |
|  |  |  |  | [6] | (results pooled across sites) **Additive effect** of herbivore exclusion (or effect of small herbivores?) on the biomass of *Deschampsia flexuosa and* *Vaccinium myrtillus and the percent cover of vegetation on disturbed plots* (control = LHe < SHe); additive effect on *V. vitis-idaea* (control <= LHe <= SHe). | 12 studies (all included in meta-regressions) |
|  |  |  |  | [7] | **Herbivore exclusion** (irrespective of which herbivore group was excluded) increased shrub biomass and shrub height (control < LHe = SHe). | 33 studies (all included in meta-regressions) |
| Abisko (Sweden) | LHe, SHe, control | 3 replicate blocks established in 1998 in moist heath (experimental design included also forest plots, not included here) | LHe: reindeer (*Rangifer tarandus*); SHe: gray-side vole (*Myodes rufocanus*), Norwegian lemming (*Lemmus lemmus*) | [7] | **No effects** of herbivore exclusion on shrub height or shrub biomass. | 20 studies (14 included in meta-regressions, 6 excluded because it was not possible to estimate effect size) |
|  |  |  |  | [4] | **Additive effect** of herbivore exclusion on LAI, vascular plant density, the abundance of evergreen shrubs and the height of dwarf shrubs (SHe > LHe = control). **No effect** on NDVI or LAI, the abundance of other plant functional groups or plant diversity. **Herbivore exclusion** (irrespective of which herbivore group was excluded) increased lichen thickness (control < LHe = SHe) and reduced the abundance of graminoids (LHe = SHe > control). | 39 studies (33 included in meta-regressions, 3 included in quantitative synthesis, 3 excluded because they referred to outcome variables reported by less than 5 articles) |
|  |  |  |  | [8] | **Additive effect** of herbivore exclusion on NDVI and on the biomass of *Betula nana* (SHe >= LHe >= control). **Herbivore exclusion** (irrespective of which herbivore group was excluded) increased total abundance of vascular plants and the biomass of *Empetrum nigrum* (control < LHe = SHe), and reduced GPP and NEE (LHe = SHe < control). | 24 studies (21 included in meta-regressions, 3 included in quantitative synthesis) |
|  |  |  |  | [9] | **Herbivore exclusion** (irrespective of which herbivore group was excluded) increased biomass of Betula nana (control < LHe = SHe). **Additive effect** of herbivore exclusion (or effect of small herbivores?) on the biomass of *Empetrum hermaphroditum*, *Vaccinium myrtillus*, *V. vitis-idaea* and the lichen *Cladina mitis* (control = LHe < SHe). | 3 studies ( 2 included in meta-regressions, 1 excluded because of lack of diversity contrast) |
|  |  |  |  | [5] | (results pooled across sites) **Additive effect** of herbivore exclusion (or effect of small herbivores?) on the relative change in abundance of *Vaccinium myrtillus*, *V. vitis-idaea, Dicranum spp.* and *Polytrichum spp* (control = LHe < SHe) and for *Ptilidium ciliare* (control = LHe > SHe). **Herbivore exclusion** (irrespective of which herbivore group was excluded) increased the relative change in abundance of *Cladina mitis* (control < LHe = SHe). | 3 studies (all included in meta-regressions) |
|  |  |  |  | [6] | (results pooled across sites) **Additive effect** of herbivore exclusion (or effect of small herbivores?) on the biomass of *Deschampsia flexuosa and* *Vaccinium myrtillus and the percent cover of vegetation on disturbed plots* (control = LHe < SHe); additive effect on *V. vitis-idaea* (control <= LHe <= SHe). | 12 studies (all included in meta-regressions) |
| Seiland (Norway) | LHe, SHe, control | 3 replicate blocks established in 1998 | LHe: reindeer (*Rangifer tarandus*); SHe: gray-side vole (*Myodes rufocanus*), Norwegian lemming (*Lemmus lemmus*) | [7] | **No effects** of herbivore exclusion on shrub height or shrub biomass. | 33 studies (24 included in meta-regressions, 9 excluded because it was not possible to estimate effect size) |
|  |  |  |  | [5] | (results pooled across sites) **Additive effect** of herbivore exclusion (or effect of small herbivores?) on the relative change in abundance of *Vaccinium myrtillus*, *V. vitis-idaea, Dicranum spp.* and *Polytrichum spp* (control = LHe < SHe) and for *Ptilidium ciliare* (control = LHe > SHe). **Herbivore exclusion** (irrespective of which herbivore group was excluded) increased the relative change in abundance of *Cladina mitis* (control < LHe = SHe). | 3 studies (all included in meta-regressions) |
|  |  |  |  | [6] | (results pooled across sites) **Additive effect** of herbivore exclusion (or effect of small herbivores?) on the biomass of *Deschampsia flexuosa and* *Vaccinium myrtillus and the percent cover of vegetation on disturbed plots* (control = LHe < SHe); additive effect on *V. vitis-idaea* (control <= LHe <= SHe). | 12 studies (all included in meta-regressions) |
| Joatka (Norway) | LHe, SHe, control | 3 replicate blocks established in 1998 in dry heath (experimental design included also forest plots, not included here) | LHe: reindeer (*Rangifer tarandus*); SHe: gray-side vole (*Myodes rufocanus*), Norwegian lemming (*Lemmus lemmus*) | [7] | **Herbivore exclusion** (irrespective of which herbivore group was excluded) increased shub height (control < LHe = SHe). | 33 studies (27 included in meta-regressions, 6 excluded because it was not possible to estimate effect size) |
|  |  |  |  | [5] | (results pooled across sites) **Additive effect** of herbivore exclusion (or effect of small herbivores?) on the relative change in abundance of *Vaccinium myrtillus*, *V. vitis-idaea, Dicranum spp.* and *Polytrichum spp* (control = LHe < SHe) and for *Ptilidium ciliare* (control = LHe > SHe). **Herbivore exclusion** (irrespective of which herbivore group was excluded) increased the relative change in abundance of *Cladina mitis* (control < LHe = SHe). | 3 studies (all included in meta-regressions) |
|  |  |  |  | [6] | (results pooled across sites) **Additive effect** of herbivore exclusion (or effect of small herbivores?) on the biomass of *Deschampsia flexuosa and* *Vaccinium myrtillus and the percent cover of vegetation on disturbed plots* (control = LHe < SHe); additive effect on *V. vitis-idaea* (control <= LHe <= SHe). | 12 studies (all included in meta-regressions) |
|  |  |  |  | [4] | **Herbivore exclusion** (irrespective of which herbivore group was excluded) increased lichen thickness (control < LHe = SHe) and reduced the abundance of graminoids (LHe = SHe > control). **No effects** of herbivore exclusion in any other measured variable. | 39 studies (33 included in meta-regressions, 3 included in quantitative synthesis, 3 excluded because they referred to outcome variables reported by less than 5 articles) |
| Finnmarksvidda (Norway) | LHe, SHe, control | 4 blocks with exclosures established in 1992 (experimental design included also fertilization) | LHe: reindeer (*Rangifer tarandus*); SHe: Norwegian lemmings (*Lemmus lemmus*), grey-sided voles (*Clethrionomys rufocanus*) | [10] | **Additive effect** of herbivore exclusion on abundance of *Vaccinium myrtillus* (SHe > LHe > control) and *Vaccinium vitis-idaea* (SHe > LHe = control). **Herbivore exclusion** (irrespective of which herbivore group was excluded) increased abundance of vascular plants and graminoids (control < LHe = SHe). | 32 studies (all included in meta-regressions) |
|  |  |  |  | [11] | **Additive effect** of herbivore exclusion on soil organic N and soil respiration (SHe > LHe > control). **Herbivore exclusion** (irrespective of which herbivore group was excluded) reduced soil moisture (LHe = SHe > control) | 72 studies (60 included in meta-regressions, 4 included in quantitative synthesis, 8 excluded because they referred to outcome variables reported by less than 5 articles) |
| Varanger Peninsula (Norway) | LHe, SHe, control | several catchments (2 in [12], 3 in [13]) with 29-39 replicates each | LHe: reindeer (*Rangifer tarandus*); SHe: Norwegian lemming (*Lemmus lemmus*), tundra vole (*Microtus oeconomus*) and grey-sided vole (*Myodes rufocanus*) | [12] | **Additive effect** of herbivore exclusion after three years on deciduous shrubs in one of the catchments and for forbs and standing dead cover in another (control = LHe < SHe). | 35 studies (all excluded because it was not possible to estimate effect size) |
|  |  |  |  | [13] | **Herbivore exclusion** (irrespective of which herbivore group was excluded) increased shoot size of Salix phylicifolia in one catchment (control < LHe = SHe) but not in others. **Additive effect** of herbivore exclusion (or effect of small herbivores?) on Salix recruit mortality in two of the catchments (control = LHe > SHe). | 12 studies (6 included in quantitative synthesis, 6 excluded because it was not possible to estimate effect size) |
| NW Finnish Lapland (Finland) | LHe, Mhe, control | 40 willow ramets at 2 sites protected by reindeer or reindeer+ptarmigan exclosures (half of the willows were experimentally rejuvenated) | LHe: reindeer (*Rangifer tarandus*); Mhe: willow ptarmigan (*Lagopus lagopus*); exclosures did not prevent access by small mammals. | [14] | Browsing by vertebrate herbivores modulated the effects of precipitation and temperature on shoot growth of tea-leaved willow (*Salix phylicifolia*). Temperature had positive effects on ramets browsed by ptarmigan but negative effects on ramets browsed by reindneer, probably due to more extensive browsing damage caused by reindeer. | 6 studies (2 included in meta-regressions, 2 in quantitative synthesis, 2 excluded because of lack of diversity contrast) |
| Yamal Peninsula (Russia) | LHe, MHe, She, control | 3 blocks with two replicates per treatment, in each of 3 habitats; exclosures established in 2014 | LHe: reindeer (*Rangifer tarandus*); MHe: willow ptarmigan (*Lagopus lagopus*) and mountain hares (*Lepus timidus*); SHe: narrow-headed voles (*Microtus gregalis*), Middendorf's voles (*Microtus middendorffii*) and collared lemmings (*Dicrostonyx torquatus*). | [15] | **Compensatory effects** on total biomass and forbs in willow meadows and trends in the same direction for grasses (control = MHe < LHe = She. | 18 studies (all excluded because it was not possible to estimate effect size) |

[1] Gough et al. 2008. Arctic, Antarctic and Alpine Research. 40: 65-73.

[2] Min et al. 2021. Environmental Research Letters. 16: 024027

[3] Roy et al. 2020. Artic, Antartic and Alpine Research. 52: 109-119.

[4] Lindén et al. 2021. Ecology and Evolution. 11: 12141–12152.

[5] Olofsson et al. 2004. Oikos. 106: 324-334.

[6] Olofsson et al. 2005. Landscape Ecology. 20: 351-359.

[7] Olofsson et al. 2009. Global Change Biology. 15: 2681-2693.

[8] Metcalfe and Olofsson 2015. Oikos. 124: 1632-1638.

[9] Olofsson et al. 2013. Philosophical Transactions of the Royal Society. 368: 20120486.

[10] Grellmann 2002. Oikos. 98: 190-204.

[11] Stark and Grellmann. 2002. Ecology. 83:2736-2744.

[12] Ravolainen et al. 2011. Basic and Applied Ecology. 12:243-653.

[13] Ravolainen et al. 2014. Journal of Applied Ecology. 51:234-241.

[14] Virtanen et al. 2021. Journal of Ecology. 109: 1250-1262.

[15] Baubin et al. 2016. Czech Polar Reports. 6:132-140.

# Study validity assessment

Consistency among reviewers in scoring study validity (856 studies) was substantial for overall score of risk of bias (89.4 % agreement, κ = 0.67). The agreement between reviewers was substantial for the risk of misclassified comparison or performance bias (criteria 3 and 4; 87.9% agreement, Cohen’s kappa κ = 0.67) and moderate for other criteria. The disparity between reviewers in scoring biases due to confounding factors (criterion 1; 76.2% agreement, κ = 0.46) mainly came from the uncertainty regarding the inclusion or exclusion of confounding factors by the studies. For example, the lack of mention of confounding variables was seen as low risk by some reviewers who assumed that researchers would mention confounding variables for their study system if they considered them relevant. The risk of post-intervention sampling bias (criterion 2) had a 76.9% agreement (κ = 0.55). Some reviewers took ambiguity in the randomness of initial site selection as cause for concern, whilst others saw the random allocation of treatments within-site sufficient to warrant low-risk status. For the risk of measurement bias (criterion 5; 75.6 % agreement; κ = 0.49), there was a discrepancy over whether unclear researcher awareness of treatments automatically warranted bias, or whether due to the robustness of methods this bias was assumed unlikely regardless of researcher awareness. The risk of outcome reporting bias (criterion 6) had 76.2% agreement (κ = 0.48). For the risk of outcome assessment biases (criterion 7; 83.1% agreement; κ = 0.44, no studies clearly stated whether the data analyst was aware of the levels of herbivore diversity, meaning all studies could be minimally classified as medium risk, but reviewers differed in their scoring of this concern.

**Figure S4.5.** Scores for study validity assessment of all studies included in the systematic review. The seven criteria follow Konno et al. (2021); criteria 3 and 4 were assessed together because they refer to either observational or experimental studies.


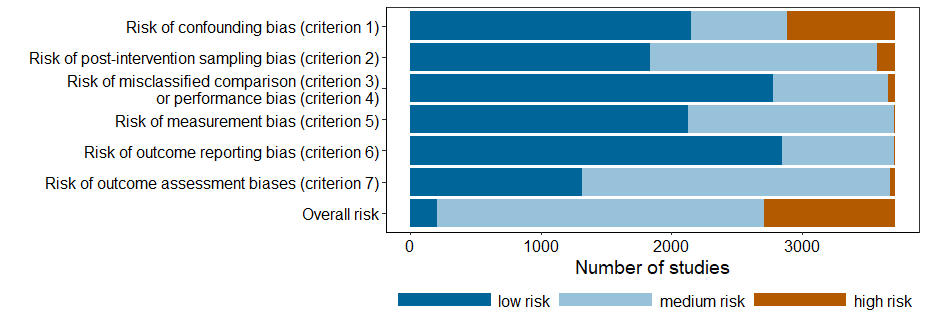


# Multi-moderator meta-regressions

We tested the effects of moderators on outcome variables reported by at least 10 articles (25 outcome variables) by comparing a model including a particular moderator to the corresponding intercept-only model. When several moderators had a significant effect on an outcome variable, we built multi-moderator meta-regression models (**Table S4.10**). We assessed model fit by comparing these multi-moderator meta-regression models to the corresponding intercept-only models with a Log-likelihood Ratio test.

**Table S4.10** Multi-moderator meta-regression models. Such models were built for 15 outcome variables reported by at least 10 articles. Values of Likelihood Ratio Test (LRT) and the associated p-value (pval), BIC, AIC, AICc and increment in AICc (deltaAICc) refer to the comparison to the intercept-only model (no moderators). Moderators indicated in bold had a significant effect on the outcome variable.

| outcome variable | formula | LRT | pval | BIC | AIC | AICc | deltaAICc |
| --- | --- | --- | --- | --- | --- | --- | --- |
| plant CN ratio | yi_smd ~ **change_f** + **exclusion** + spatial_resolution | 13.29 | 0.01 | 132.51 | 121.62 | 125.77 | -1.91 |
| plant C content | yi_smd ~ **overall_criterion** | 8.61 | 0.0135 | 295.88 | 283.78 | 284.56 | -4.13 |
| abundance bryophytes | yi_smd ~ exclusion | 3.98 | 0.046 | 406.30 | 395.22 | 395.57 | -1.84 |
| abundance dwarf shrubs | yi_smd ~ herb_fgr_change + exclusion + spatial resolution | 19.26 | 0.0017 | 650.17 | 623.58 | 624.32 | -8.65 |
| abundance graminoids | yi_smd ~ **change_f** | 15.80 | 0.0033 | 915.78 | 891.44 | 891.93 | -7.41 |
| abundance lichens | yi_smd ~ **change_f** | 10.65 | 0.0138 | 490.77 | 472.63 | 473.21 | -4.23 |
| abundance tall shrubs | yi_smd ~ **error_type** | 8.12 | 0.0436 | 140.07 | 128.97 | 131.07 | -0.58 |
| plant diversity | yi_smd ~ distance_to_treeline + **habitat_type** | 11.51 | 0.0093 | 137.09 | 124.43 | 125.98 | -4.37 |
| plant fitness | yi_smd ~ **error_type** | 6.12 | 0.0133 | 271.75 | 263.05 | 263.72 | -3.85 |
| plant height | yi_smd ~ **change_f** + **error_type** + exclusion + habitat_type + overall_criterion + **study_length** | 42.74 | <0.001 | 299.68 | 260.61 | 266.32 | -13.27 |
| plant productivity | yi_smd ~ **permafrost_D** + recent_warming + temperature | 11.45 | 0.0095 | 170.61 | 158.15 | 159.76 | -4.27 |
| plant species richness | yi_smd ~ permafrost_D | 3.91 | 0.0481 | 132.82 | 123.60 | 124.18 | -1.67 |
| plant structure | yi_smd ~ **year.c** | 5.11 | 0.0238 | 371.00 | 360.62 | 361.04 | -2.93 |
| soil C labile | yi_smd ~ error_type | 7.25 | 0.0266 | 90.81 | 85.14 | 88.67 | -0.99 |
| soil moisture | yi_smd ~ **error_type** + **year.c** | 18.33 | <0.001 | 111.09 | 102.29 | 105.65 | -9.83 |

We checked the robustness of the results of the multi-moderator meta-regression models to the presence of influential studies (sensitivity analyses). We calculated Cook’s distance using the ‘leave-one-out’ approach recommended by (Nakagawa et al., 2023), which estimates the Mahalanobis distance between the average effects predicted by the models with and without a particular study. We considered values of Cook’s distance >1 to be influential studies (Dhakal, 2017). Only one study (200_c) was identified as influential in the model for plant C content (**Figure S4.6**). The model without this study indicated a non-significant effect of the overall risk of bias (LRT = 1.33, p = 0.51).

**Figure S4.6** Cook’s distance for the studies included in the model for plant C content (n = 83 studies). Values of Cook’s distance >1 (orange) indicate influential studies.


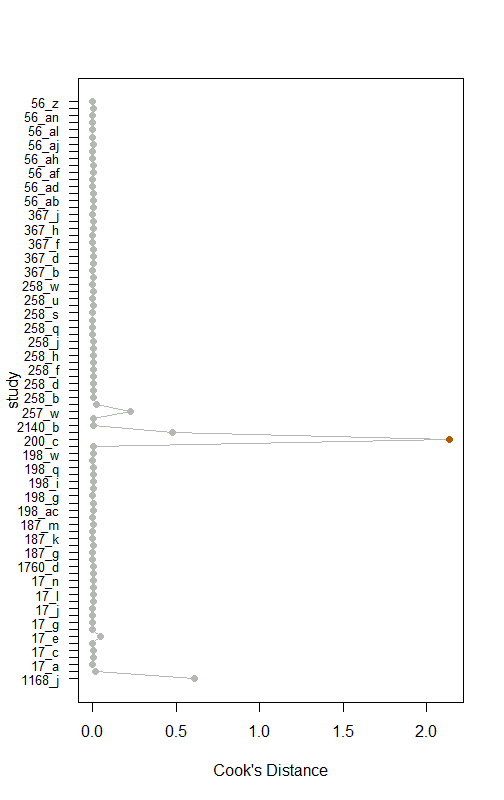

Supplement: Supplementary file 4 — Additional file 4: Extended methods and results. [file 13750_2024_330_MOESM4_ESM.docx]
