# Supplementary material for: Herbivore diversity effects on Arctic tundra ecosystems: a systematic review
Source: Environ Evid. 2024 Mar 25;13:6. doi: 10.1186/s13750-024-00330-9 (PMC11378771; doi:10.1186/s13750-024-00330-9)
Supplement: Supplementary file 5 — Additional file 5: Interactive map server. [file 13750_2024_330_MOESM5_ESM.docx]

Additional file 5: Interactive map server: https://shiny.vm.ntnu.no/users/speed/ArcticHerbivoreDiversitySystematicReview/
